# Exploring Molecular Mechanism of Fluoxetine in Animal Models of Depression via Integrated Metabolomic and Proteomic Analysis

**DOI:** 10.1111/jcmm.71112

**Published:** 2026-03-25

**Authors:** Yin Chen, Wei Tang, Xiangkun Tao, Chi Liu, Hailin Wu, Ning Wang, Cenyu Liao, Nanxi He, Yiwen Chen, Yiyun Liu, Dongfang Wang, Siwen Gui, Xiaogang Zhong, Yuan Liu, Bin Hua, Lining Yang, Juncai Pu, Peng Xie

**Affiliations:** ^1^ Department of Neurology, NHC Key Laboratory of Diagnosis and Treatment on Brain Functional Diseases The First Affiliated Hospital of Chongqing Medical University Chongqing China; ^2^ Chongqing Medical University Chongqing China; ^3^ The Jinfeng Laboratory Chongqing China; ^4^ Department of Rehabilitation Medicine, Key Laboratory of Physical Medicine and Precision Rehabilitation of Chongqing Municipal Health Commission The First Affiliated Hospital of Chongqing Medical University Chongqing China; ^5^ Chongqing Institute for Brain and Intelligence Chongqing China

**Keywords:** antidepressant, fluoxetine, metabolomics, proteomics

## Abstract

Fluoxetine is a widely used antidepressant, yet integrated analyses of its molecular mechanism remain limited. This study systematically investigated potential molecular mechanisms underlying the antidepressant effects of fluoxetine by integrating these scattered data. Using the ProMENDA database, we identified metabolites and proteins altered by fluoxetine in the brain of animal models of depression. We curated 273 differentially expressed metabolite entries and 791 differentially expressed protein entries from fluoxetine treatment and performed vote‐counting, pathway enrichment, pathway crosstalk and drug‐associated metabolite set enrichment analyses. Vote‐counting analysis showed altered neurotransmitter levels, including increased levels of monoamines and decreased levels of neurotoxic quinolinic acid and glutamate. The results of pathway analyses based on both altered metabolites and proteins showed 121 significantly enriched pathways. Pathway crosstalk analysis identified four pathway‐based modules, which were mainly involved in amino acid metabolism, neurotransmitters and multiple biological processes. Drug‐associated metabolite set enrichment analysis revealed 76 significantly enriched drug‐related pathways, which were mainly involved in antidepressants. This study provides a comprehensive understanding of the antidepressant effects of fluoxetine, which may provide insights for the development of novel antidepressants.

## 1 Introduction

Depression, a prevalent psychiatric disorder, ranks as one of the leading etiological factors contributing to the global burden of disease ([1] GBD 2021 US Burden of Disease Collaborators). It is also noted that depression is commonly comorbid in patients with physical illness [2]. Selective serotonin reuptake inhibitors (SSRIs), such as fluoxetine, are among the most widely prescribed antidepressants in the US [3]. Prior meta‐analyses demonstrated the efficacy of fluoxetine in paediatric and adult patients with depression [4, 5]. Despite decades of progress in understanding the neurobiological mechanism of fluoxetine, its therapeutic mechanism remains incompletely elucidated. Thus, further exploration of its molecular basis is still needed.

Multi‐omics analysis serves as a powerful approach for unravelling the molecular mechanisms of complex diseases from a systems biology perspective [6, 7]. Among omics methodologies, metabolomics demonstrates high precision in quantifying metabolite levels, including neurotransmitters, amino acids and lipids, while proteomics enables systematic analysis of protein expression in a convenient way [8, 9]; given that metabolites and proteins are essential for cellular functions in diseases, metabolomic and proteomic techniques have been extensively employed to investigate the molecular mechanism of fluoxetine. A previous metabolomic analysis showed that myo‐inositol was a potential biomarker for the therapeutic efficacy of fluoxetine treatment in the hippocampus of a chronic unpredictable mild stress model of depression [10]. Another proteomic analysis revealed that fluoxetine administration could modulate cytoskeletal dynamics, energy metabolism, neuronal plasticity and calcium homeostasis in the subcellular compartments of the prefrontal cortex [11]. Although numerous studies investigated molecular changes resulting from fluoxetine treatment based on metabolomic and proteomic techniques, these individual studies could not identify the full range of molecular alterations due to methodological limitations. There remains a lack of systematic analysis to reveal the overall molecular mechanism associated with fluoxetine treatment. Integrating metabolomic and proteomic data from a big‐data perspective can elucidate the molecular mechanism underlying fluoxetine. Integrated analysis that combines different levels of molecular data could clarify how proteins and metabolites mediate the antidepressant effect of fluoxetine. Up to now, a systematic understanding of these molecular alterations is still lacking.

The aim of this study was to conduct a systematic analysis to integrate metabolomic and proteomic changes induced by fluoxetine treatment in animal models of depression. Therefore, we collected these omics data related to fluoxetine intervention in the brain of animal models of depression and explored potential biological functions and interactions from a pathway‐based perspective. This study allowed us to elucidate how fluoxetine exerted antidepressant effects by altering metabolomic and proteomic profiles in depression.

## 2 Materials and Methods

### 2.1 Data Source

The data used in this study were sourced from the ProMENDA database (https://menda.cqmu.edu.cn). Detailed descriptions of this resource have been provided in our prior work [12, 13]. Briefly, we retrieved relevant metabolomic and proteomic studies from databases including PubMed, Embase, Web of Science and PsycINFO. After screening titles and abstracts, full‐text articles were reviewed and data were extracted for the construction of the ProMENDA database. This database provides comprehensive descriptions of differential metabolites and proteins, covering both molecular‐level and study‐level data, such as molecule IDs (protein names, UniProt IDs and gene symbols for protein data; metabolite names, HMDB IDs, KEGG IDs and PubChem IDs for metabolite data), experimental design, biological species, depression subtypes, tissue types, analytical techniques and citations. To date, the database contains 43,366 molecular entries, including 22,519 differential metabolite entries and 20,847 differential protein entries, with 18,190 metabolite entries and 17,868 protein entries from animal models.

### 2.2 Data Selection

To identify candidate data to be included, the following steps were used. Studies that investigated metabolomic and proteomic changes associated with fluoxetine treatment were included. Specifically, only rodent models (e.g., mice and rats) were chosen because they provided the most extensive and consistent data, allowing for more reliable pathway analyses. We excluded data from human studies to minimise confounding factors arising from interspecies differences, especially given that there were only dozens of molecular entries from human studies. We also excluded studies that explored molecular alterations between treatment response and non‐response groups where all subjects received fluoxetine, as these studies focused on variability in treatment outcomes. Only studies that used brain samples were included, as the brain is the primary organ of action for fluoxetine. Data from peripheral tissues (e.g., blood, liver) were excluded due to both limited availability in the database and potential differences in molecular mechanism compared to the central nervous system. Following this screening process, we compiled the proteomic and metabolomic datasets associated with fluoxetine treatment, respectively.

### 2.3 Analytical Strategy

To assess the overall dysregulated trends of candidate molecules across studies, we employed a vote‐counting approach. This method counted how many studies found a molecule statistically significant, followed by scoring its dysregulation frequency. Based on the dysregulation trend for each molecule in the proteomic and metabolomic datasets, a scoring system was implemented where each molecule in each study was assigned a score of “1” for significant up‐regulation or “‐1” for significant down‐regulation. The sum of the scores was then calculated for each molecule. A positive sum indicated a consistent trend of up‐regulation across the studies, while a negative sum indicated the opposite. This approach has been widely adopted in our previous studies, as it is the most feasible method when raw data of all molecules were unavailable [8, 14].

**FIGURE 1:**
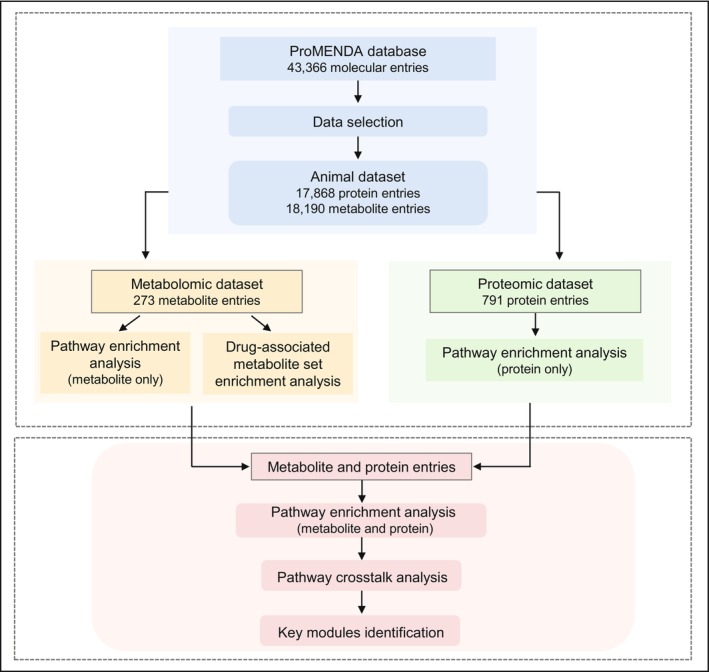
The flowchart for the current study.

The following biological analyses were performed to elucidate biological functions of identified metabolites and proteins. At the metabolite level, pathway enrichment analysis and drug‐associated metabolite set enrichment analysis were conducted based on all curated metabolites. At the protein level, pathway enrichment analysis was applied. To identify key pathways potentially implicated in both metabolite and protein levels, an integrated pathway enrichment analysis based on both metabolites and proteins was also performed. To explore potential interplays among significantly enriched pathways, we also conducted pathway crosstalk analysis. The flowchart for biological analyses is shown in Figure 1.

### 2.4 Biological Analysis

#### 2.4.1 Pathway Enrichment Analysis

Pathway enrichment analysis was performed to gain mechanistic insight into molecule lists generated from these omics datasets. Candidate metabolite or protein lists were uploaded to MetaboAnalyst 6.0 (https://www.metaboanalyst.ca), followed by pathway enrichment analysis using default parameters [15]. At the metabolite level, metabolite pathway analysis was performed with the upload of HMDB IDs for metabolites. At the protein and integrated metabolite‐protein levels, the analyses were conducted with the upload of both HMDB IDs for metabolites and gene symbols for proteins, using the ‘all pathways (gene only)’ and ‘all pathways (integrated)’ databases from the joint pathway analysis, respectively. The remaining parameters were set as follows: enrichment method, hypergeometric test; organism, *Mus musculus* . The significance of enriched pathways was assessed based on the Kyoto Encyclopedia of Genes and Genomes (KEGG) pathways via Fisher's exact test and *p‐*values were adjusted using the Benjamini‐Hochberg procedure. Pathways with false discovery rates (FDR) < 0.05 were defined as significantly enriched.

#### 2.4.2 Drug‐Associated Metabolite Set Enrichment Analysis

To examine the relationship between candidate metabolites and other pharmacological agents, we performed drug‐associated metabolite set enrichment analysis using MetaboAnalyst. Using this approach, we assessed whether the uploaded metabolites showed significant enrichment in any of the 461 predefined drug‐related metabolite sets. Pathway significance was evaluated using Fisher's exact test. Pathways with FDR < 0.05 were defined as statistically significantly enriched.

#### 2.4.3 Pathway Crosstalk Analysis

Pathway crosstalk analysis examined shared molecules across enriched pathways, based on the hypothesis that more shared molecules indicated stronger crosstalk between any pair of pathways. We used the R software (version 4.5.0) to evaluate the number of shared molecules between pathways. Interacted pathways were required to share at least three differential molecules [16]. Based on these potential interactions between pathways, a pathway‐based network was constructed. Network visualisation was performed using Gephi (version 0.10.1, https://gephi.org) [17]. To identify core biological functions of this network, we also analysed the hub pathway modules. Potential modules were identified using the MCODE plug‐in in Cytoscape (version 3.10.3) with the following filter setting: degree cut‐off = 2; node score cut‐off = 0.2; k‐core = 3; and max depth = 100.

## 3 Results

### 3.1 Overview of Datasets

**FIGURE 2:**
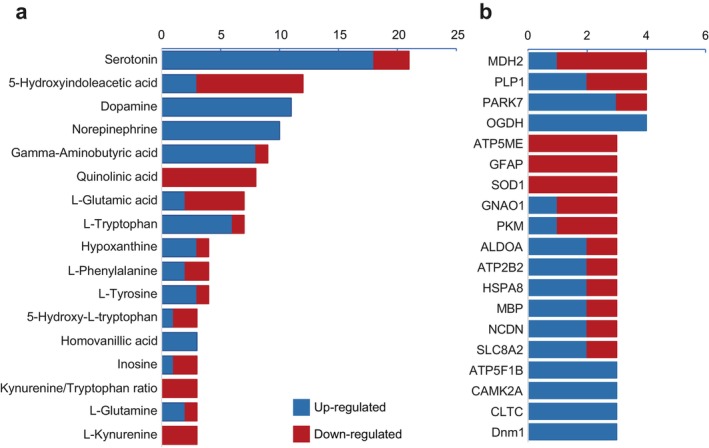
Vote‐counting results for altered metabolites and proteins. (a) The altered trends of differential metabolites; (b) The altered trends of differential proteins. Molecules reported at least three times were counted. Blue represents an increasing trend, and red represents a decreasing trend.

At the metabolite level, we included 273 differential metabolites from 35 studies in the ProMENDA database. Numbers of excluded molecular entries were summarised in Table S1. Following removal of duplicates, 152 unique metabolites were identified (Table S2). Among these metabolites, serotonin, 5‐hydroxyindoleacetic acid, dopamine and norepinephrine were reported in more than 10 studies. Vote‐counting analyses identified serotonin, norepinephrine and dopamine as the top‐ranked consistently up‐regulated metabolites and 5‐hydroxyindoleacetic acid, quinolinic acid and glutamic acid as the top‐ranked down‐regulated metabolites (Figure 2a).

At the protein level, we included 791 differentially expressed protein entries from nine studies. The reasons for excluding protein entries were summarised in Table S1. After removing duplicates, we obtained 646 unique proteins (Table S3). Four proteins were reported in at least three studies, with OGDH and PARK7 showing an increasing trend and MDH2 showing a decreasing trend (Figure 2b).

### 3.2 Functional Analysis Based on Molecules Associated With Fluoxetine Intervention

#### 3.2.1 Metabolite Level

**FIGURE 3:**
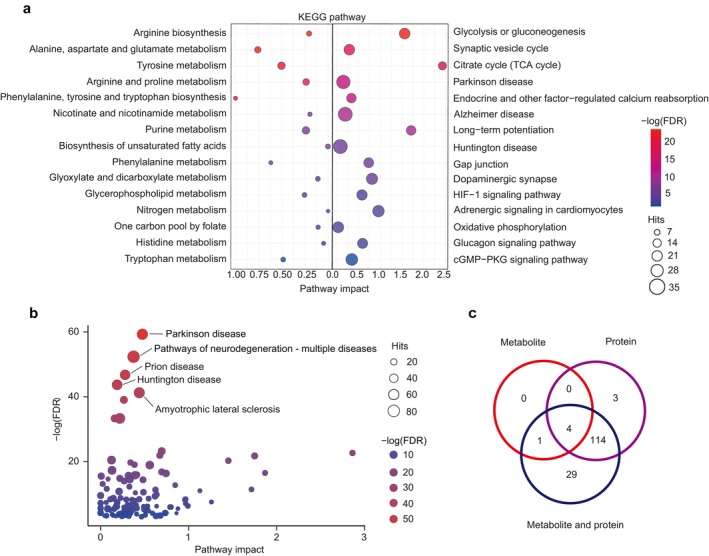
Pathways significantly enriched in each analysis. (a) Pathway enrichment results based on metabolite data (left) and protein data (right), respectively. Pathway names are displayed along the y‐axis, while the x‐axis represents the pathway impact, and the colour represents the statistical significance. (b) Results of joint pathway analysis based on both metabolite and protein data. The statistical significance is represented by the y‐axis position and colour of the bubbles, while the x‐axis represents the pathway impact. (c) Numbers of shared pathways in metabolite and protein data.

Based on the metabolomic data, the results of the pathway enrichment analysis showed that 15 pathways were enriched at a significance threshold of *p* < 0.05. After multiple testing correction, five pathways were significantly enriched (FDR < 0.05; Figure 3a). These significant pathways included ‘arginine biosynthesis’, ‘alanine, aspartate and glutamate metabolism’, ‘tyrosine metabolism’, ‘arginine and proline metabolism’ and ‘phenylalanine, tyrosine and tryptophan biosynthesis’.

We also performed drug‐associated metabolite set enrichment analysis. The results showed that 76 drug‐related pathways were significantly enriched (FDR < 0.05; Table S4). As expected, these pathways involved antidepressants such as escitalopram, desipramine, fluoxetine and imipramine. Moreover, our results revealed enrichment in pathways including the ‘antibiotic pathway’, ‘analgesic and anaesthetic drug pathways’ and ‘purine metabolism pathway’, suggesting that fluoxetine may influence these biological processes.

#### 3.2.2 Protein Level

Based on the proteomic data, the pathway enrichment analysis identified 121 significantly altered pathways (FDR < 0.05). The top‐ranked pathways included ‘glycolysis/gluconeogenesis’, ‘synaptic vesicle cycle’, ‘tricarboxylic acid (TCA) cycle’, ‘endocrine and other factor‐regulated calcium reabsorption’ and ‘Parkinson disease’ (Figure 3a).

### 3.3 Joint Pathway Analysis Based on Both Metabolite and Protein Data

**FIGURE 4:**
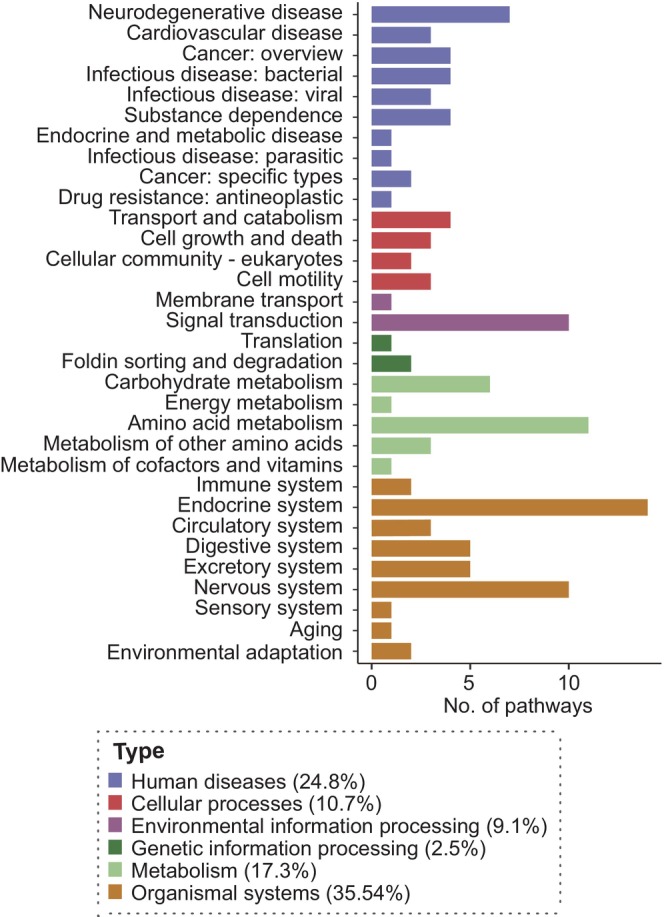
Classification of significantly enriched pathways identified based on the KEGG database.

To integrate system‐level changes based on both metabolomic and proteomic data, joint pathway analysis was performed. The results revealed 121 KEGG pathways were significantly enriched (FDR < 0.05; Figure 3b). The top 5 significantly altered pathways were Parkinson disease’, ‘pathways of neurodegeneration—multiple diseases', ‘prion disease’, ‘Huntington disease’ and ‘amyotrophic lateral sclerosis'. Based on the KEGG database, these pathways were mainly involved in ‘organismal systems' (43 pathways), especially for ‘endocrine system’ (14 pathways) and ‘nervous system’ (10 pathways). These pathways were also involved in ‘human diseases' (30 pathways), especially for ‘neurodegenerative disease’ (7 pathways). ‘Metabolism’ was also enriched (22 pathways), especially for ‘amino acid metabolism’ (11 pathways) and ‘carbohydrate metabolism’ (six pathways; Figure 4).

Among these pathways, four pathways, including ‘arginine biosynthesis’, ‘alanine, aspartate and glutamate metabolism’, ‘tyrosine metabolism’ and ‘arginine and proline metabolism’, were also enriched in pathways based on both metabolomic and proteomic data (Figure 3c).

### 3.4 Pathway Crosstalk Analysis

**FIGURE 5:**
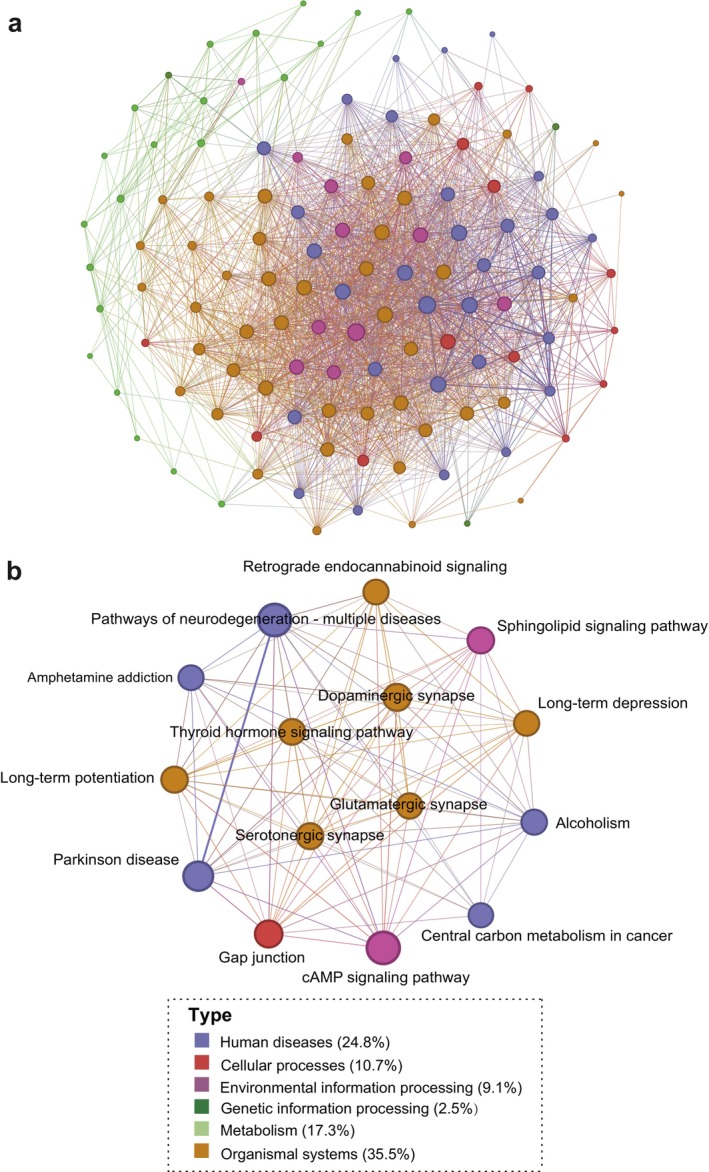
The results of pathway crosstalk analysis. (a) The results of pathway crosstalk analysis based on significantly enriched pathways. (b) Top 15 pathways with the most connections to surrounding nodes.

**FIGURE 6:**
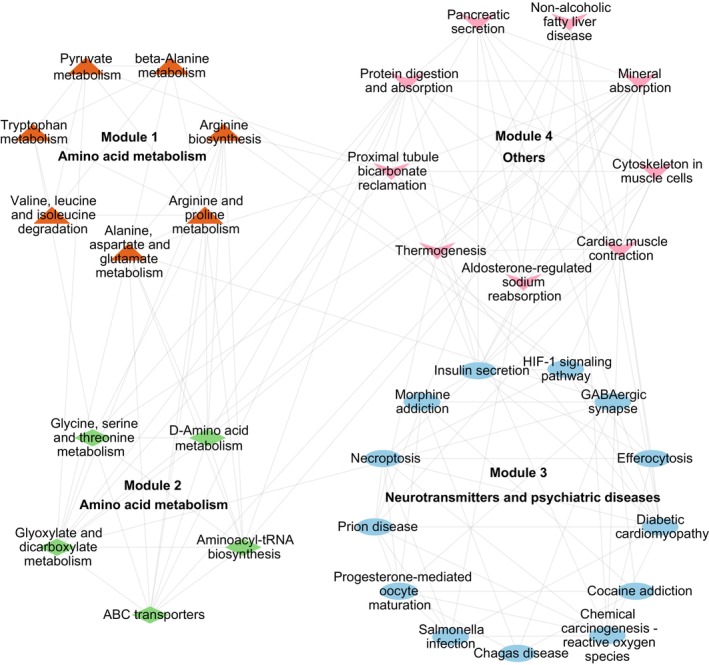
Pathway‐based modules identified by MCODE analysis.

To investigate functional interactions among enriched pathways, we performed crosstalk analysis based on 121 enriched pathways. There were 2305 pairs of pathways that shared at least three molecules. As shown in Figure 5a, a network that revealed the potential interactions between pathways was constructed. In this network, nodes present cross‐talked pathways, node colours represent their KEGG functional categories and node sizes indicate the numbers of connected pathways, with larger nodes representing greater connectivity. The numbers of candidate molecules that overlap between two cross‐talked pathways are indicated by the width of the edges. Among them, the top‐ranked pathways with more cross‐talked pathways are shown in Figure 5b. We then divided these pathways into pathway‐based modules using the MCODE analysis. The results showed that four modules were identified. The main biological function of the first module was amino acid metabolism, which contained seven pathways, including ‘tryptophan metabolism’, ‘alanine, aspartate and glutamate metabolism’ and ‘arginine and proline metabolism’. The second module was also mainly involved in amino acid metabolism, which contained five pathways, such as ‘D‐amino acid metabolism’, ‘glycine, serine and threonine metabolism’ and ‘aminoacyl‐tRNA biosynthesis’. The third module was mainly involved in neurotransmitters and psychiatric diseases, which contained 13 pathways, including ‘GABAergic synapse’, ‘morphine addiction’ and ‘cocaine addiction’. The fourth module was mainly involved in multiple biological processes, which contained nine pathways, including ‘thermogenesis’, ‘mineral absorption’ and ‘protein digestion and absorption’ (Figure 6).

## 4 Discussion

Although a large number of metabolomic and proteomic studies have revealed molecular alterations induced by fluoxetine in animal models of depression, a systematic integration of these findings to provide a comprehensive view is still lacking. Here, we employed an integrated approach to elucidate the biological functions associated with fluoxetine based on 273 metabolite entries and 791 protein entries. Our results showed that these molecules were mainly involved in four pathway‐based modules, including amino acid metabolism, neurotransmitters and multiple biological processes. Our findings revealed the effect of fluoxetine on metabolomic and proteomic profiles in the brain of animal models of depression, enhancing our understanding of its biological functions.

The most obvious finding to emerge from the current study is that two pathway‐based modules were mainly involved in amino acid metabolism, which contained seven and five pathways, respectively. Among these pathways, alanine, aspartate and glutamate metabolism were consistently enriched at metabolite and protein levels, separately or combined, suggesting the important role of glutamate metabolism. Vote‐counting analysis also revealed decreased glutamate levels in the brain after fluoxetine treatment. These results were consistent with data obtained in patients with depression [18]. Excessive release of glutamate, the central excitatory neurotransmitter, may induce postsynaptic neuronal calcium overload and neuronal atrophy [19]. Previous research showed that fluoxetine mitigated excitotoxicity and improved synaptic plasticity by suppressing glutamatergic neuronal activity or enhancing astrocytic glutamate reuptake [20]. These results agreed with the findings of other studies, which revealed the important role of novel glutamate‐targeted antidepressants [21, 22]. Notably, two recently FDA‐approved antidepressants, esketamine and dextromethorphan‐bupropion, target the glutamate system. Their approval confirmed the importance of glutamate pathway in depression, which marked a breakthrough by providing new treatment options, especially for patients unresponsive to conventional therapies [22]. Moreover, alterations were observed in arginine metabolism. This finding was in keeping with previous clinical studies, which reported abnormal concentrations of arginine metabolites in mood disorders [23]. Fluoxetine may modulate this pathway by inhibiting nitric oxide synthesis, reducing neuroinflammation and enhancing synaptic plasticity [24, 25]. Modulating arginine metabolism offers a promising strategy for developing new depression treatments [26]. Beyond these specific amino acids, we also observed that fluoxetine influenced aminoacyl‐RNA biosynthesis, suggesting a potential role in promoting protein synthesis. Overall, these findings indicated that fluoxetine may exert antidepressant effects by modulating amino acid metabolism.

Another important finding is that the third pathway‐based module was mainly involved in neurotransmitters, which contained ‘GABAergic synapse’, ‘morphine addiction’ and ‘cocaine addiction’. The enrichment of GABAergic synapse suggested brain GABAergic signalling may serve as effective targets for treating depression [27, 28]. These results are in agreement with those obtained in previous clinical studies, which showed reduced GABA neurotransmission in depression, and fluoxetine could modulate GABA neurotransmission in opposing ways [29]. These results underscored GABAergic signalling as a promising target for developing alternative antidepressant strategies. In accordance with the results of pathway crosstalk analysis, the results of the vote‐counting analysis showed the consistently altered levels of neurotransmitters, including increased levels of monoamines and decreased levels of neurotoxic quinolinic acid and glutamate. These results confirmed the robust effect of fluoxetine in modulating monoaminergic neurotransmission [30]. It is noted that other research showed that fluoxetine, but not ketamine, reduced the production of neurotoxic quinolinic acid, which indicated that the modulation of neurotoxicity may not contribute to the rapid‐onset antidepressant effect [31]. Other clinical studies also reported that the reduction in neurotoxic quinolinic acid may serve as a useful biomarker for assessing treatment safety in patients receiving ketamine and behavioural therapy [32, 33]. Therefore, modulating neurotransmitters was essential for the therapeutic mechanism of fluoxetine.

The fourth pathway‐based module was implicated in multiple biological processes, including protein digestion and absorption, mineral absorption, aldosterone‐regulated sodium reabsorption, cardiac muscle contraction, thermogenesis, pancreatic secretion and proximal tubule bicarbonate reclamation. Although these pathways may not directly link to depression, these pathways are crucial for maintaining systemic homeostasis, including normal physiological functions of the brain [34, 35]. Our findings suggested fluoxetine may affect multiple biological processes, including influencing protein and mineral intake, pancreatic secretion and myocardial activity. This may explain why antidepressants represented by fluoxetine can lead to various side effects, such as alterations in body weight, heart rate and blood pressure [36]. In line with our findings, recent research showed that fluoxetine increased body weight by acutely suppressing the activity of a subset of brainstem serotonergic neurons and chronically reducing hypothalamic pro‐opiomelanocortin production [37]. Therefore, these findings highlight the need to compare the biological mechanisms underlying the side effects of different antidepressants, which is essential for developing new drugs with improved tolerability profiles.

It should be noted that this study had several limitations. First, the included data were restricted to rodent models; validation with human data is therefore needed to confirm the generalisability of our results. Second, the absence of transcriptomic or epigenomic datasets in the ProMENDA database limited our ability to elucidate the whole molecular landscape of fluoxetine. To address this, we plan to incorporate transcriptomic data into the ProMENDA database in subsequent work. Additionally, the biological significance of certain pathways required experimental validation. Despite these limitations, our work systematically consolidated currently recognised metabolomic and proteomic alterations induced by fluoxetine, providing foundational evidence for deciphering its therapeutic mechanisms in depression treatment.

In summary, this study set out to gain a better understanding of metabolomic and proteomic alterations of fluoxetine. This study has identified four pathway‐based modules, which are mainly involved in amino acid metabolism, neurotransmitters and multiple biological processes. These findings contribute to our understanding of molecular alterations of fluoxetine from a systems biology perspective, which could provide valuable insights into the development of novel antidepressants.

## Author Contributions

Conception and design and interpretation of data: Juncai Pu and Peng Xie. Acquisition and analysis of data: Juncai Pu, Yin Chen, Yiyun Liu, Wei Tang, Xiangkun Tao, Hailin Wu, Chi Liu, Ning Wang, Siwen Gui, Xiaogang Zhong, Dongfang Wang, Cenyu Liao, Nanxi He, Yiwen Chen, Bin Hua, Yuan Liu, Lining Yang. Drafting display items: Yin Chen, Juncai Pu, Yiyun Liu, Wei Tang, Cenyu Liao. Drafting and revising the manuscript: Juncai Pu, Yin Chen, Xiangkun Tao, Wei Tang and Peng Xie. All authors approved the final version of the manuscript.

## Funding

This work was supported by The Joint project of Chongqing Municipal Science and Technology Bureau and Chongqing Health Commission (2023CCXM003), National Natural Science Foundation of China (82371526), the Natural Science Foundation of Chongqing (CSTB2024NSCQ‐QCXMX0033, CSTB2024NSCQ‐MSX1027), National Natural Science Foundation of China (W2511089), The Chongqing Technology Innovation and Application Development key project (CSTB2024TIAD‐KPX0035), The Chongqing Science and Health Joint Medical Research Youth Project (2023QNXM035), The Fund of Moutaintop plan project in the First Affiliated Hospital of Chongqing Medical University (cyyy‐xkdfjh‐lcyj‐202301), and The Chongqing Medical Science and Technology Innovation Four Centers Construction Project—Chongqing Clinical Evaluation and Research Center for Cardiovascular and Cerebrovascular Drug and Devices (Materials) Project.

## Ethics Statement

All methods were performed in accordance with the relevant guidelines and regulations. The study was approved by The Ethics Committee of Chongqing Medical University (IACUC‐CQMU‐2024‐0115).

## Consent

The datasets generated in the current study were collected from publicly available literature or reports; therefore informed consent forms are not applicable.

## Conflicts of Interest

The authors declare no conflicts of interest.

## Supporting information


**Table S1:** Number of excluded molecular entries from the ProMENDA database
**Table S2:**. List of differential metabolite entries included in this study
**Table S3:** List of differential protein entries included in this study
**Table S4:** The results of drug‐associated metabolite set enrichment analysis

## Data Availability

The data that support the findings of this study are openly available in ProMENDA at https://menda.cqmu.edu.cn.
