# Supplementary material for: Exploring Molecular Mechanism of Fluoxetine in Animal Models of Depression via Integrated Metabolomic and Proteomic Analysis
Source: J Cell Mol Med. 2026 Mar 25;30(6):e71112. doi: 10.1111/jcmm.71112 (PMC13098112; doi:10.1111/jcmm.71112)
Supplement: Supplementary file 1 — Table S1: Number of excluded molecular entries from the ProMENDA database Table S2:. List of differential metabolite entries included in this study Table S3: List of differential protein entries included in this study Table S4: The results of drug‐associated metabolite set enrichment analysis [file JCMM-30-e71112-s001.docx]

**Article**

[***Journal of Cellular and Molecular Medicine***](https://sci.justscience.cn/details.html?sci=1&id=632)

**Exploring molecular mechanism of fluoxetine in animal models of depression via integrated metabolomic and proteomic analysis**

Yin Chen ^1^, Wei Tang ^1^, Xiangkun Tao ^2^, Chi Liu ^1^, Hailin Wu ^1^, Ning Wang^1^, Cenyu Liao ^2^, Nanxi He ^2^, Yiwen Chen ^2^, Yiyun Liu ^1^, Dongfang Wang ^1^, Siwen Gui ^1^, Xiaogang Zhong ^1^, Yuan Liu ^3^, Bin Hua ^1^, Lining Yang ^4^, Juncai Pu ^1,*^, Peng Xie ^1,3,4,5,*^

^1^ Department of Neurology, NHC Key Laboratory of Diagnosis and Treatment on Brain Functional Diseases, The First Affiliated Hospital of Chongqing Medical University, Chongqing, China

^2^ Chongqing Medical University, Chongqing, China

^3^ The Jinfeng Laboratory, Chongqing, China

^4^ Department of Rehabilitation Medicine, Key Laboratory of Physical Medicine and Precision Rehabilitation of Chongqing Municipal Health Commission, The First Affiliated Hospital of Chongqing Medical University, Chongqing, China

^5^ Chongqing Institute for Brain and Intelligence, Chongqing, China

^*^ Correspondence to:

Dr. Juncai Pu (pujuncai0910@163.com),

and Prof. Peng Xie (xiepeng@cqmu.edu.cn)

Department of Neurology, NHC Key Laboratory of Diagnosis and Treatment on Brain Functional Diseases, The First Affiliated Hospital of Chongqing Medical University, No.1 Youyi Road, Yuzhong District, Chongqing, 400016, China.

**Supplementary information**

**Supplemental Tables**

**Supplemental Table 1.** Number of excluded molecular entries from the ProMENDA database ...................................................................................................................................2

**Supplemental Table 2**. List of differential metabolite entries included in this study …………. 3

**Supplemental Table 3.** List of differential protein entries included in this study.....................13

**Supplemental Table 4.** The results of drug-associated metabolite set enrichment analysis..42

| **Supplemental Table 1.** Number of excluded molecular entries from the ProMENDA database. | | |
| --- | --- | --- |
| **Exclusion reasons** | **No of metabolite entries** | **No of protein entries** |
| Other type studies | 16350 | 17443 |
| Other drug studies | 3320 | 1871 |
| Other tissue studies | 1812 | 470 |
| Patient studies | 751 | 272 |
| Non-human primate studies | 10 | 0 |
| Other drug studies (in combination) | 3 | 0 |
| **Total** | **22246** | **20056** |

| **Supplemental Table 2.** List of differential metabolite entries included in this study. | | | |
| --- | --- | --- | --- |
| **Metabolite name** | **HMDB** | **KEGG** | **PubChem** |
| 5-Hydroxyindoleacetic acid | HMDB0000763 | C05635 | 1826 |
| 5-HIAA/5-HT ratio | N/A | N/A | N/A |
| Creatine | HMDB0000064 | C00300 | 586 |
| Niacinamide | HMDB0001406 | C00153 | 936 |
| Hypoxanthine | HMDB0000157 | C00262 | 790 |
| L-Phenylalanine | HMDB0000159 | C00079 | 6140 |
| Indoleacrylic acid | HMDB0000734 | N/A | 15030923 |
| (9Z-Hexadecanoyl)-glycerol | N/A | N/A | N/A |
| Docosanoyl-glycerol | N/A | N/A | N/A |
| 3-Hydroxyhexadecanoic acid | HMDB0061658 | N/A | N/A |
| LysoPC(16:0) | N/A | C04230 | N/A |
| D-Glucurono-6,3-lactone | HMDB0006355 | C02670 | 2724333 |
| Inosine | HMDB0000195 | C00294 | 6021 |
| Glycine | HMDB0000123 | C00037 | 750 |
| L-Leucine | HMDB0000687 | C00123 | 6106 |
| L-Malic acid | HMDB0000156 | C00149 | 222656 |
| Palmitic acid | HMDB0000220 | C00249 | 985 |
| Stearic acid | HMDB0000827 | C01530 | 5281 |
| Creatinine | HMDB0000562 | C00791 | 588 |
| L-Glutamic acid | HMDB0000148 | C00025 | 33032 |
| N-Acetyl-L-aspartic acid | HMDB0000812 | C01042 | 65065 |
| myo-Inositol | HMDB0000211 | C00137 | 892 |
| Adenosine | HMDB0000050 | C00212 | 60961 |
| N-Acetyl-L-aspartic acid | HMDB0000812 | C01042 | 65065 |
| Phosphorylcholine | HMDB0001565 | C00588 | 1014 |
| Ascorbic acid | HMDB0000044 | C00072 | 54670067 |
| Taurine | HMDB0000251 | C00245 | 1123 |
| Hypoxanthine | HMDB0000157 | C00262 | 790 |
| Phosphoenolpyruvic acid | HMDB0000263 | C00074 | 1005 |
| L-Glutamine | HMDB0000641 | C00064 | 5961 |
| L-Leucine | HMDB0000687 | C00123 | 6106 |
| L-Phenylalanine | HMDB0000159 | C00079 | 6140 |
| LysoPC(16:0) | N/A | C04230 | N/A |
| Glutathione | HMDB0000125 | C00051 | 124886 |
| L-Tyrosine | HMDB0000158 | C00082 | 6057 |
| L-Tryptophan | HMDB0000929 | C00078 | 6305 |
| Indoleacrylic acid | HMDB0000734 | N/A | 15030923 |
| L-Arginine | HMDB0000517 | C00062 | 6322 |
| Phenylpyruvic acid | HMDB0000205 | C00166 | 997 |
| Inosine | HMDB0000195 | C00294 | 6021 |
| L-Threonine | HMDB0000167 | C00188 | 6288 |
| Serotonin | HMDB0000259 | C00780 | 5202 |
| Norepinephrine | HMDB0000216 | C00547 | 439260 |
| Quinolinic acid | HMDB0000232 | C03722 | 1066 |
| 5-Hydroxy-L-tryptophan | HMDB0000472 | C00643 | 144 |
| Serotonin | HMDB0000259 | C00780 | 5202 |
| 5-Hydroxyindoleacetic acid | HMDB0000763 | C05635 | 1826 |
| Quinolinic acid | HMDB0000232 | C03722 | 1066 |
| 5-Hydroxyindoleacetic acid | HMDB0000763 | C05635 | 1826 |
| Quinolinic acid | HMDB0000232 | C03722 | 1066 |
| 5-Hydroxyindoleacetic acid | HMDB0000763 | C05635 | 1826 |
| Quinolinic acid | HMDB0000232 | C03722 | 1066 |
| 5-Hydroxyindoleacetic acid | HMDB0000763 | C05635 | 1826 |
| Quinolinic acid | HMDB0000232 | C03722 | 1066 |
| Serotonin | HMDB0000259 | C00780 | 5202 |
| 5-Hydroxyindoleacetic acid | HMDB0000763 | C05635 | 1826 |
| Quinolinic acid | HMDB0000232 | C03722 | 1066 |
| 5-Hydroxy-L-tryptophan | HMDB0000472 | C00643 | 144 |
| Serotonin | HMDB0000259 | C00780 | 5202 |
| 5-Hydroxyindoleacetic acid | HMDB0000763 | C05635 | 1826 |
| Quinolinic acid | HMDB0000232 | C03722 | 1066 |
| 5-Hydroxyindoleacetic acid | HMDB0000763 | C05635 | 1826 |
| Progesterone | HMDB0001830 | C00410 | 5994 |
| Allopregnanolone | HMDB0001449 | C13712 | 92786 |
| Pregnenolone | HMDB0000253 | C01953 | 53477678 |
| Aminoadipic acid | HMDB0000510 | C00956 | 92136 |
| L-Tryptophan | HMDB0000929 | C00078 | 6305 |
| Serotonin | HMDB0000259 | C00780 | 5202 |
| L-Kynurenine | HMDB0000684 | C00328 | 161166 |
| Kynurenine/Tryptophan ratio | N/A | N/A | N/A |
| L-Glutamine | HMDB0000641 | C00064 | 5961 |
| L-Glutamic acid | HMDB0000148 | C00025 | 33032 |
| Glutamate/Glutamine ratio | N/A | N/A | N/A |
| Anserine | HMDB0000194 | C01262 | 112072 |
| Aminoadipic acid | HMDB0000510 | C00956 | 92136 |
| Isobutyryl-L-carnitine | HMDB0000736 | N/A | 168379 |
| L-Cystine | HMDB0000192 | C00491 | 67678 |
| L-Glutamine | HMDB0000641 | C00064 | 5961 |
| 4-Hydroxyproline | HMDB0000725 | C01157 | 5810 |
| Serotonin | HMDB0000259 | C00780 | 5202 |
| Dopamine | HMDB0000073 | C03758 | 681 |
| Norepinephrine | HMDB0000216 | C00547 | 439260 |
| 5-Hydroxyindoleacetic acid | HMDB0000763 | C05635 | 1826 |
| Gamma-Aminobutyric acid | HMDB0000112 | C00334 | 119 |
| Norepinephrine | HMDB0000216 | C00547 | 439260 |
| Serotonin | HMDB0000259 | C00780 | 5202 |
| Gamma-Aminobutyric acid | HMDB0000112 | C00334 | 119 |
| 5-Hydroxyindoleacetic acid | HMDB0000763 | C05635 | 1826 |
| L-Glutamic acid | HMDB0000148 | C00025 | 33032 |
| Gamma-Aminobutyric acid | HMDB0000112 | C00334 | 119 |
| L-Glutamic acid | HMDB0000148 | C00025 | 33032 |
| Methoxyepinephrine | N/A | N/A | N/A |
| 3-Methoxytyramine | HMDB0000022 | C05587 | 1669 |
| L-Tryptophan | HMDB0000929 | C00078 | 6305 |
| L-Tyrosine | HMDB0000158 | C00082 | 6057 |
| Homovanillic acid | HMDB0000118 | C05582 | 1738 |
| Gamma-Aminobutyric acid | HMDB0000112 | C00334 | 119 |
| DOPA | HMDB0000181 | C00355 | 6047 |
| Methoxyepinephrine | N/A | N/A | N/A |
| Acetylcholine | HMDB0000895 | C01996 | 187 |
| 3-Methoxytyramine | HMDB0000022 | C05587 | 1669 |
| Homovanillic acid | HMDB0000118 | C05582 | 1738 |
| Epinephrine | HMDB0000068 | C00788 | 5816 |
| Acetylcholine | HMDB0000895 | C01996 | 187 |
| L-Tryptophan | HMDB0000929 | C00078 | 6305 |
| L-Tyrosine | HMDB0000158 | C00082 | 6057 |
| N-Formyl-L-glutamic acid | HMDB0003470 | C01045 | 439376 |
| Inosinic acid | HMDB0000175 | C00130 | 8582 |
| Glutathione | HMDB0000125 | C00051 | 124886 |
| Inosine | HMDB0000195 | C00294 | 6021 |
| LysoPE(18:1/0:0) | N/A | N/A | N/A |
| Oleamide | HMDB0002117 | C19670 | 5283387 |
| (9S,10S)-10-hydroxy-9-(phosphonooxy)octadecanoate | HMDB0059632 | C15989 | 23724633 |
| LysoPC(18:0) | N/A | C04230 | N/A |
| Oleic acid | HMDB0000207 | C00712 | 445639 |
| MG(18:0/0:0/0:0) | HMDB0011131 | C13856 | 15560610 |
| PE(20:3/P-18:1) | N/A | C00350 | N/A |
| PC(18:0/22:1) | N/A | C00157 | N/A |
| Phosphatidic acid | N/A | C00416 | N/A |
| Acyl carnitines | N/A | N/A | N/A |
| Sphingomyelin | N/A | C00550 | N/A |
| PC(36:4p)+H | N/A | C00157 | N/A |
| PA(18:0/18:1)-H | N/A | C00416 | N/A |
| PA(18:0/22:6)-H | N/A | C00416 | N/A |
| Phosphatidylinositol | N/A | C01194 | N/A |
| PA(46:7)-H | N/A | C00416 | N/A |
| Phosphatidylethanolamine | HMDB0060501 | C00350 | 17754131 |
| Diglyceride | N/A | C00165 | N/A |
| Triglyceride | N/A | C00422 | N/A |
| Monoglyceride | N/A | C01885 | N/A |
| PS(41:1)-H | N/A | C02737 | N/A |
| PE(34:1e)+H | N/A | C00350 | N/A |
| PE(18:0p/20:1)+H | N/A | C00350 | N/A |
| PE(16:0/20:4)-H | N/A | C00350 | N/A |
| PE(18:0/18:2)-H | N/A | C00350 | N/A |
| PE(16:0e/22:6)-H | N/A | C00350 | N/A |
| PE(18:0p/22:5)-H | N/A | C00350 | N/A |
| PC(42:1)+H | N/A | C00157 | N/A |
| CerG1(d18:2/24:0+O)+H | N/A | N/A | N/A |
| Glucosylceramides | N/A | N/A | N/A |
| Diglyceride | N/A | C00165 | N/A |
| PS(39:0)-H | N/A | C02737 | N/A |
| PE(18:1p/20:1)+H | N/A | C00350 | N/A |
| PE(16:0/20:4)-H | N/A | C00350 | N/A |
| PE(18:0/18:2)-H | N/A | C00350 | N/A |
| PE(16:0p/22:5)-H | N/A | C00350 | N/A |
| PE(18:1/20:4)-H | N/A | C00350 | N/A |
| PE(18:0/20:4)-H | N/A | C00350 | N/A |
| PE(18:1p/22:6)-H | N/A | C00350 | N/A |
| PE(18:0p/22:4)-H | N/A | C00350 | N/A |
| PC(33:0)+H | N/A | C00157 | N/A |
| PC(36:1e)+H | N/A | C00157 | N/A |
| PC(36:0)+H | N/A | C00157 | N/A |
| PC(38:4)+H | N/A | C00157 | N/A |
| PC(18:0/20:4)+H | N/A | C00157 | N/A |
| PC(38:1)+H | N/A | C00157 | N/A |
| PC(16:0/16:0)+HCOO | N/A | C00157 | N/A |
| PC(16:0e/18:1)+HCOO | N/A | C00157 | N/A |
| PC(18:0p/22:1)+HCOO | N/A | C00157 | N/A |
| Cer(d18:2/18:0)+HCOO | N/A | C00195 | N/A |
| Dopamine | HMDB0000073 | C03758 | 681 |
| Serotonin | HMDB0000259 | C00780 | 5202 |
| Norepinephrine | HMDB0000216 | C00547 | 439260 |
| Gamma-Aminobutyric acid | HMDB0000112 | C00334 | 119 |
| Dopamine | HMDB0000073 | C03758 | 681 |
| L-Glutamic acid | HMDB0000148 | C00025 | 33032 |
| Dopamine | HMDB0000073 | C03758 | 681 |
| Norepinephrine | HMDB0000216 | C00547 | 439260 |
| Gamma-Aminobutyric acid | HMDB0000112 | C00334 | 119 |
| 5-Hydroxytryptophol | HMDB0001855 | N/A | 9061 |
| L-Glutamic acid | HMDB0000148 | C00025 | 33032 |
| Gamma-Aminobutyric acid | HMDB0000112 | C00334 | 119 |
| Docosahexaenoic acid | HMDB0002183 | C06429 | 445580 |
| Oleoylethanolamide | HMDB0002088 | C20792 | 5283454 |
| Eicosapentaenoic acid | HMDB0001999 | C06428 | 446284 |
| 20-HDoHE | HMDB0060048 | N/A | 131769807 |
| 4-HDoHE | HMDB0060049 | N/A | 131769808 |
| Resolvin D1 | HMDB0003733 | C18178 | 16061135 |
| Arachidonic acid | HMDB0001043 | C00219 | 444899 |
| 12-HETE | HMDB0006111 | C14777 | 5283155 |
| 15-HETE | HMDB0003876 | C04742 | 5280724 |
| 5S,15S-DiHETE | HMDB0010216 | N/A | 5283158 |
| 8-iso prostaglandin F2 | N/A | N/A | 160 |
| 8-iso-PGA1 | HMDB0002236 | N/A | 6473771 |
| Serotonin | HMDB0000259 | C00780 | 5202 |
| Serotonin | HMDB0000259 | C00780 | 5202 |
| S-Adenosylmethionine | HMDB0001185 | C00019 | 16757548 |
| Nicotinic acid | HMDB0001488 | C00253 | 938 |
| Adenosine monophosphate | HMDB0000045 | C00020 | 6083 |
| L-Tryptophan | HMDB0000929 | C00078 | 6305 |
| 1-Pyrroline-5-carboxylic acid | HMDB0001301 | C03912 | 440162 |
| Oleoylethanolamide | HMDB0002088 | C20792 | 5283454 |
| Quinolinic acid | HMDB0000232 | C03722 | 1066 |
| L-Cystine | HMDB0000192 | C00491 | 67678 |
| Kynurenine/Tryptophan ratio | N/A | N/A | N/A |
| Serotonin | HMDB0000259 | C00780 | 5202 |
| Dopamine | HMDB0000073 | C03758 | 681 |
| Dopamine | HMDB0000073 | C03758 | 681 |
| Serotonin | HMDB0000259 | C00780 | 5202 |
| Norepinephrine | HMDB0000216 | C00547 | 439260 |
| Dopamine | HMDB0000073 | C03758 | 681 |
| Norepinephrine | HMDB0000216 | C00547 | 439260 |
| Serotonin | HMDB0000259 | C00780 | 5202 |
| Norepinephrine/Vanylglycol ratio | N/A | N/A | N/A |
| Serotonin/5-Hydroxyindoleacetic acid ratio | N/A | N/A | N/A |
| Dopamine | HMDB0000073 | C03758 | 681 |
| Norepinephrine | HMDB0000216 | C00547 | 439260 |
| Serotonin | HMDB0000259 | C00780 | 5202 |
| 3,4-Dihydroxybenzeneacetic acid/Dopamine ratio | N/A | N/A | N/A |
| Serotonin | HMDB0000259 | C00780 | 5202 |
| Serotonin | HMDB0000259 | C00780 | 5202 |
| 5-Hydroxyindoleacetic acid | HMDB0000763 | C05635 | 1826 |
| 5-HIAA/5-HT ratio | N/A | N/A | N/A |
| Serotonin | HMDB0000259 | C00780 | 5202 |
| Dopamine | HMDB0000073 | C03758 | 681 |
| 3,4-Dihydroxybenzeneacetic acid | HMDB0001336 | C01161 | 547 |
| Serotonin | HMDB0000259 | C00780 | 5202 |
| Dopamine | HMDB0000073 | C03758 | 681 |
| 3,4-Dihydroxybenzeneacetic acid | HMDB0001336 | C01161 | 547 |
| Homovanillic acid | HMDB0000118 | C05582 | 1738 |
| Serotonin | HMDB0000259 | C00780 | 5202 |
| L-Kynurenine | HMDB0000684 | C00328 | 161166 |
| L-Phenylalanine | HMDB0000159 | C00079 | 6140 |
| L-Tryptophan | HMDB0000929 | C00078 | 6305 |
| N-Acetylaspartylglutamic acid | HMDB0001067 | C12270 | 5255 |
| Gamma-Aminobutyric acid | HMDB0000112 | C00334 | 119 |
| Epinephrine | HMDB0000068 | C00788 | 5816 |
| Norepinephrine | HMDB0000216 | C00547 | 439260 |
| L-Proline | HMDB0000162 | C00148 | 145742 |
| L-Phenylalanine | HMDB0000159 | C00079 | 6140 |
| L-Kynurenine | HMDB0000684 | C00328 | 161166 |
| Kynurenine/Tryptophan ratio | N/A | N/A | N/A |
| 5-Hydroxy-L-tryptophan | HMDB0000472 | C00643 | 144 |
| Oleamide | HMDB0002117 | C19670 | 5283387 |
| Anandamide | HMDB0004080 | C11695 | 5281969 |
| Norepinephrine | HMDB0000216 | C00547 | 439260 |
| Dopamine | HMDB0000073 | C03758 | 681 |
| Gamma-Aminobutyric acid | HMDB0000112 | C00334 | 119 |
| Palmitic acid | HMDB0000220 | C00249 | 985 |
| Stearic acid | HMDB0000827 | C01530 | 5281 |
| Oleic acid | HMDB0000207 | C00712 | 445639 |
| Arachidonic acid | HMDB0001043 | C00219 | 444899 |
| Fumaric acid | HMDB0000134 | C00122 | 444972 |
| L-Proline | HMDB0000162 | C00148 | 145742 |
| L-Serine | HMDB0000187 | C00065 | 5951 |
| Hypoxanthine | HMDB0000157 | C00262 | 790 |
| Uracil | HMDB0000300 | C00106 | 1174 |
| L-Lysine | HMDB0000182 | C00047 | 5962 |
| L-Aspartic acid | HMDB0000191 | C00049 | 5960 |
| Urea | HMDB0000294 | C00086 | 1176 |
| L-Tyrosine | HMDB0000158 | C00082 | 6057 |
| Hypoxanthine | HMDB0000157 | C00262 | 790 |
| Sphingosine | HMDB0000252 | C00319 | 5353955 |
| L-Tryptophan | HMDB0000929 | C00078 | 6305 |
| GlcCer(d18:1/24:1) | HMDB0004975 | C01190 | 20057357 |
| Threonic acid | HMDB0000943 | C01620 | 151152 |
| PS(18:0/22:6(4Z,7Z,10Z,13Z,16Z,19Z)) | HMDB0010167 | C02737 | 24779546 |
| 5b-cyprinol sulfate | HMDB0006888 | C05468 | 53477904 |
| L-Glutamic acid | HMDB0000148 | C00025 | 33032 |
| Xanthine | HMDB0000292 | C00385 | 1188 |
| PC(24:1(15Z)/24:1(15Z)) | HMDB0008816 | C00157 | 6452499 |
| Enkephalin L | HMDB0001045 | N/A | 461776 |
| PC(15:0/18:1(11Z)) | HMDB0007938 | C00157 | 24778662 |
| PE(18:1(11Z)/22:4(7Z,10Z,13Z,16Z)) | HMDB0009042 | C00350 | 53479640 |
| D-Sedoheptulose 7-phosphate | HMDB0001068 | C05382 | 22833559 |
| Hypotaurine | HMDB0000965 | C00519 | 107812 |
| L-Acetylcarnitine | HMDB0000201 | C02571 | 7045767 |
| Hexanoylcarnitine | HMDB0000756 | N/A | 3246938 |
| Xanthosine | HMDB0000299 | C01762 | 64959 |
| Riboflavin | HMDB0000244 | C00255 | 493570 |
| Serotonin | HMDB0000259 | C00780 | 5202 |
| 5-Hydroxyindoleacetic acid | HMDB0000763 | C05635 | 1826 |
| Serotonin | HMDB0000259 | C00780 | 5202 |
| Abbreviations: LysoPC, lysophosphatidylcholine; LysoPE, lysophosphatidylethanolamine;MG, monoacylglycerol; PA, phosphatidic acid; PC, phosphatidylcholine; PE, phosphatidylethylethanolamine; PS, phosphatidylserine. | | | |

| **Supplemental Table 3.** List of differential protein entries included in this study. | | |
| --- | --- | --- |
| **UniProt entry** | **Protein name** | **Gene symbol** |
| CATD_RAT | Cathepsin D | Ctsd |
| STMN1_RAT | Stathmin | Stmn1 |
| DYN1_RAT | Dynamin-1 | Dnm1 |
| PARK7_RAT | Parkinson disease protein 7 homolog | Park7 |
| ALDOA_RAT | Fructose-bisphosphate aldolase A | Aldoa |
| ARPC5_RAT | Actin-related protein 2/3 complex subunit 5 | Arpc5 |
| HSP7C_RAT | Heat shock cognate 71 kDa protein | Hspa8 |
| GAMT_RAT | Guanidinoacetate N-methyltransferase | Gamt |
| PHB1_RAT | Prohibitin 1 | Phb1 |
| HOME1_RAT | Homer protein homolog 1 | Homer1 |
| G3P_RAT | Glyceraldehyde-3-phosphate dehydrogenase | Gapdh |
| UBE2N_RAT | Ubiquitin-conjugating enzyme E2 N | Ube2n |
| MIF_RAT | Macrophage migration inhibitory factor | Mif |
| KAD4_RAT | Adenylate kinase 4, mitochondrial | Ak4 |
| NDUV2_RAT | NADH dehydrogenase [ubiquinone] flavoprotein 2, mitochondrial | Ndufv2 |
| RL40_RAT | Ubiquitin-ribosomal protein eL40 fusion protein | Uba52 |
| CX6A1_RAT | Cytochrome c oxidase subunit 6A1, mitochondrial | Cox6a1 |
| PPIA_RAT | Peptidyl-prolyl cis-trans isomerase A | Ppia |
| ATP5I_RAT | ATP synthase subunit e, mitochondrial | Atp5me |
| CYC_RAT | Cytochrome c, somatic | Cycs |
| ODP2_RAT | Dihydrolipoyllysine-residue acetyltransferase component of pyruvate dehydrogenase complex, mitochondrial | Dlat |
| DOPD_RAT | D-dopachrome decarboxylase | Ddt |
| MAAI_RAT | Maleylacetoacetate isomerase | Gstz1 |
| SODM_RAT | Superoxide dismutase [Mn], mitochondrial | Sod2 |
| GSTP1_RAT | Glutathione S-transferase P | Gstp1 |
| NDK7_RAT | Nucleoside diphosphate kinase 7 | Nme7 |
| PHKG2_RAT | Phosphorylase b kinase gamma catalytic chain, liver/testis isoform | Phkg2 |
| B3GL1_RAT | UDP-GalNAc:beta-1,3-N-acetylgalactosaminyltransferase 1 | B3galnt1 |
| MAIP1_RAT | m-AAA protease-interacting protein 1, mitochondrial | Maip1 |
| B5DF57_RAT | protein-glutamine gamma-glutamyltransferase | Epb42 |
| PRRT2_RAT | Proline-rich transmembrane protein 2 | Prrt2 |
| MMSA_RAT | Methylmalonate-semialdehyde dehydrogenase [acylating], mitochondrial | Aldh6a1 |
| CIAO3_RAT | Cytosolic iron-sulfur assembly component 3 | Ciao3 |
| IGS21_RAT | Immunoglobulin superfamily member 21 | Igsf21 |
| MOES_RAT | Moesin | Msn |
| SHPS1_RAT | Tyrosine-protein phosphatase non-receptor type substrate 1 | Sirpa |
| F264_RAT | 6-phosphofructo-2-kinase/fructose-2,6-bisphosphatase 4 | Pfkfb4 |
| DNJB9_RAT | DnaJ homolog subfamily B member 9 | Dnajb9 |
| PCP4_RAT | Calmodulin regulator protein PCP4 | Pcp4 |
| SMAG1_RAT | Protein Smaug homolog 1 | Samd4a |
| BID_RAT | BH3-interacting domain death agonist | Bid |
| D4ACA3_RAT | Sister chromatid cohesion protein DCC1 | Dscc1 |
| SEC13_RAT | Protein SEC13 homolog | Sec13 |
| IT70B_RAT | Intraflagellar transport protein 70B | Ift70b |
| NEUM_RAT | Neuromodulin | Gap43 |
| G3V7B8_RAT | ERCC excision repair 8, CSA ubiquitin ligase complex subunit | Ercc8 |
| TEKT4_RAT | Tektin-4 | Tekt4 |
| OFUT1_RAT | GDP-fucose protein O-fucosyltransferase 1 | Pofut1 |
| VPS36_RAT | Vacuolar protein-sorting-associated protein 36 | Vps36 |
| TBA1C_RAT | Tubulin alpha-1C chain | Tuba1c |
| ATGA1_RAT | Autophagy-related protein 101 | Atg101 |
| CAN9_RAT | Calpain-9 | Capn9 |
| VAPA_RAT | Vesicle-associated membrane protein-associated protein A | Vapa |
| B1WC73_RAT | ADP-ribosylation factor-like protein 6 | Arl6 |
| CALM1_RAT | Calmodulin-1 | Calm1 |
| ROA1_RAT | Heterogeneous nuclear ribonucleoprotein A1 | Hnrnpa1 |
| RBM43_RAT | RNA-binding protein 43 | Rbm43 |
| GIMD1_RAT | GTPase IMAP family member GIMD1 | Gimd1 |
| HMOX2_RAT | Heme oxygenase 2 | Hmox2 |
| MBP_RAT | Myelin basic protein | Mbp |
| CABP1_RAT | Calcium-binding protein 1 | Cabp1 |
| PHF7_RAT | PHD finger protein 7 | Phf7 |
| A0A0G2JW59_RAT | receptor protein-tyrosine kinase | Flt3 |
| KIF28_RAT | Kinesin-like protein KIF28P | Kif28p |
| KCIP4_RAT | Kv channel-interacting protein 4 | Kcnip4 |
| MUSK_RAT | Muscle, skeletal receptor tyrosine protein kinase | Musk |
| B2RYL6_RAT | Odf2l protein | Odf2l |
| ECHD1_RAT | Ethylmalonyl-CoA decarboxylase | Echdc1 |
| KLHL2_RAT | Kelch-like protein 2 | Klhl2 |
| A0A096MJD8_RAT | SH2 domain containing 5 | Sh2d5 |
| PRS54_RAT | Inactive serine protease 54 | Prss54 |
| MAG_RAT | Myelin-associated glycoprotein | Mag |
| D4A8H1_RAT | RELB proto-oncogene, NF-kB subunit | Relb |
| ZN382_RAT | Zinc finger protein 382 | Znf382 |
| D3ZLA3_RAT | Copine 3 | Cpne3 |
| NU2M_RAT | NADH-ubiquinone oxidoreductase chain 2 | mt-Nd2 |
| Q6IFV6_RAT | Keratin 35 | Krt35 |
| DERPC_RAT | Decreased expression in renal and prostate cancer protein | Derpc |
| CEP63_RAT | Centrosomal protein of 63 kDa | Cep63 |
| PIM3_RAT | Serine/threonine-protein kinase pim-3 | Pim3 |
| FAKD4_RAT | FAST kinase domain-containing protein 4 | Tbrg4 |
| C1GLC_RAT | C1GALT1-specific chaperone 1 | C1galt1c1 |
| PLPP_RAT | Chronophin | Pdxp |
| PSD13_RAT | 26S proteasome non-ATPase regulatory subunit 13 | Psmd13 |
| FAKD2_RAT | FAST kinase domain-containing protein 2, mitochondrial | Fastkd2 |
| TRIM2_RAT | Tripartite motif-containing protein 2 | Trim2 |
| TESK2_RAT | Dual specificity testis-specific protein kinase 2 | Tesk2 |
| SPA3K_RAT | Serine protease inhibitor A3K | Serpina3k |
| STPG1_RAT | O(6)-methylguanine-induced apoptosis 2 | Stpg1 |
| CDV3_RAT | Protein CDV3 homolog | Cdv3 |
| D3ZR21_RAT | NTPase, KAP family P-loop domain containing 1 | Nkpd1 |
| SMAD7_RAT | Mothers against decapentaplegic homolog 7 | Smad7 |
| DPF1_RAT | Zinc finger protein neuro-d4 | Dpf1 |
| FURIN_RAT | Furin | Furin |
| KLC1_RAT | Kinesin light chain 1 | Klc1 |
| FUND1_RAT | FUN14 domain-containing protein 1 | Fundc1 |
| CATD_RAT | Cathepsin D | Ctsd |
| PALLD_RAT | Palladin | Palld |
| COQ8B_RAT | Atypical kinase COQ8B, mitochondrial | Coq8b |
| F1LXV3_RAT | Protein kinase domain-containing protein | Stk26 |
| EPDR1_RAT | Mammalian ependymin-related protein 1 | Epdr1 |
| ETFA_RAT | Electron transfer flavoprotein subunit alpha, mitochondrial | Etfa |
| FXR1_RAT | RNA-binding protein FXR1 | Fxr1 |
| KCC2D_RAT | Calcium/calmodulin-dependent protein kinase type II subunit delta | Camk2d |
| G3V923_RAT | SRY-box transcription factor 17 | Sox17 |
| ATPK_RAT | ATP synthase subunit f, mitochondrial | Atp5mf |
| PARK7_RAT | Parkinson disease protein 7 homolog | Park7 |
| SAM50_RAT | Sorting and assembly machinery component 50 homolog | Samm50 |
| IMA5_RAT | Importin subunit alpha-5 | Kpna1 |
| PSB5_RAT | Proteasome subunit beta type-5 | Psmb5 |
| 6PGL_RAT | 6-phosphogluconolactonase | Pgls |
| D3Z9E6_RAT | Cleavage and polyadenylation specificity factor subunit 2 | Cpsf2 |
| H2AY_RAT | Core histone macro-H2A.1 | Macroh2a1 |
| GPDA_RAT | Glycerol-3-phosphate dehydrogenase [NAD(+)], cytoplasmic | Gpd1 |
| BST1_RAT | ADP-ribosyl cyclase/cyclic ADP-ribose hydrolase 2 | Bst1 |
| CAN5_RAT | Calpain-5 | Capn5 |
| CLIC2_RAT | Chloride intracellular channel protein 2 | Clic2 |
| ERLN2_RAT | Erlin-2 | Erlin2 |
| OTOF_RAT | Otoferlin | Otof |
| Q8R2E7_RAT | FAS-associated death domain protein | Fadd |
| CPPED_RAT | Serine/threonine-protein phosphatase CPPED1 | Cpped1 |
| D4ACL2_RAT | Tetratricopeptide repeat protein 38 | Ttc38 |
| RAB1A_RAT | Ras-related protein Rab-1A | Rab1A |
| RAB8B_RAT | Ras-related protein Rab-8B | Rab8b |
| NUD18_RAT | 8-oxo-dGDP phosphatase NUDT18 | Nudt18 |
| PI3R4_RAT | Phosphoinositide 3-kinase regulatory subunit 4 | Pik3r4 |
| GRIA1_RAT | Glutamate receptor 1 | Gria1 |
| HEM2_RAT | Delta-aminolevulinic acid dehydratase | Alad |
| B0BN47_RAT | Glutathione S-transferase | Gstm6 |
| ACSM4_RAT | Acyl-coenzyme A synthetase ACSM4, mitochondrial | Acsm4 |
| TRI63_RAT | E3 ubiquitin-protein ligase TRIM63 | Trim63 |
| KCC2A_RAT | Calcium/calmodulin-dependent protein kinase type II subunit alpha | Camk2a |
| RLA1_RAT | Large ribosomal subunit protein P1 | Rplp1 |
| KCC2B_RAT | Calcium/calmodulin-dependent protein kinase type II subunit beta | Camk2b |
| IF4A3_RAT | Eukaryotic initiation factor 4A-III | Eif4a3 |
| PI42B_RAT | Phosphatidylinositol 5-phosphate 4-kinase type-2 beta | Pip4k2b |
| NAR2A_RAT | T-cell ecto-ADP-ribosyltransferase 1 | Art2a |
| ENTP2_RAT | Ectonucleoside triphosphate diphosphohydrolase 2 | Entpd2 |
| SEPT3_RAT | Neuronal-specific septin-3 | Septin3 |
| AT2B2_RAT | Plasma membrane calcium-transporting ATPase 2 | Atp2b2 |
| D3Z956_RAT | Retinaldehyde binding protein 1 | Rlbp1 |
| KPB1_RAT | Phosphorylase b kinase regulatory subunit alpha, skeletal muscle isoform | Phka1 |
| CBPE_RAT | Carboxypeptidase E | Cpe |
| TBA1A_RAT | Tubulin alpha-1A chain | Tuba1a |
| MPC2_RAT | Mitochondrial pyruvate carrier 2 | Mpc2 |
| HNRPQ_RAT | Heterogeneous nuclear ribonucleoprotein Q | Syncrip |
| TMOD2_RAT | Tropomodulin-2 | Tmod2 |
| UBX11_RAT | UBX domain-containing protein 11 | Ubxn11 |
| UBP48_RAT | Ubiquitin carboxyl-terminal hydrolase 48 | Usp48 |
| PDIA6_RAT | Protein disulfide-isomerase A6 | Pdia6 |
| IF5A1_RAT | Eukaryotic translation initiation factor 5A-1 | Eif5a |
| MPPB_RAT | Mitochondrial-processing peptidase subunit beta | Pmpcb |
| AT1A3_RAT | Sodium/potassium-transporting ATPase subunit alpha-3 | Atp1a3 |
| NEB2_RAT | Neurabin-2 | Ppp1r9b |
| D3ZNY3_RAT | Metabolism of cobalamin associated A | Mmaa |
| CBS_RAT | Cystathionine beta-synthase | Cbs |
| NAA35_RAT | N-alpha-acetyltransferase 35, NatC auxiliary subunit | Naa35 |
| KCNH2_RAT | Potassium voltage-gated channel subfamily H member 2 | Kcnh2 |
| K1C19_RAT | Keratin, type I cytoskeletal 19 | Krt19 |
| DDX1_RAT | ATP-dependent RNA helicase DDX1 | Ddx1 |
| OTUB1_RAT | Ubiquitin thioesterase OTUB1 | Otub1 |
| TAGL2_RAT | Transgelin-2 | Tagln2 |
| RAB4B_RAT | Ras-related protein Rab-4B | Rab4b |
| PYC_RAT | Pyruvate carboxylase, mitochondrial | Pc |
| NDE1_RAT | Nuclear distribution protein nudE homolog 1 | Nde1 |
| MK10_RAT | Mitogen-activated protein kinase 10 | Mapk10 |
| B2RZ90_RAT | Pcgf5 protein | Pcgf5 |
| RAB18_RAT | Ras-related protein Rab-18 | Rab18 |
| HPCL4_RAT | Hippocalcin-like protein 4 | Hpcal4 |
| TBA8_RAT | Tubulin alpha-8 chain | Tuba8 |
| TRXR1_RAT | Thioredoxin reductase 1, cytoplasmic | Txnrd1 |
| HMGN5_RAT | High mobility group nucleosome-binding domain-containing protein 5 | Hmgn5 |
| G3V8Z6_RAT | KRIT1, ankyrin repeat containing | Krit1 |
| GNAZ_RAT | Guanine nucleotide-binding protein G(z) subunit alpha | Gnaz |
| G3PT_RAT | Glyceraldehyde-3-phosphate dehydrogenase, testis-specific | Gapdhs |
| APOA4_RAT | Apolipoprotein A-IV | Apoa4 |
| EAA4_RAT | Excitatory amino acid transporter 4 | Slc1a6 |
| Q6YDN8_RAT | GID complex subunit 8 homolog | Gid8 |
| CAPS1_RAT | Calcium-dependent secretion activator 1 | Cadps |
| D4AA42_RAT | G protein subunit alpha transducin 2 | Gnat2 |
| B2RZ65_RAT | NTF2-related export protein | Nxt1 |
| PGTA_RAT | Geranylgeranyl transferase type-2 subunit alpha | Rabggta |
| COR1A_RAT | Coronin-1A | Coro1a |
| PP1G_RAT | Serine/threonine-protein phosphatase PP1-gamma catalytic subunit | Ppp1cc |
| CNRP1_RAT | CB1 cannabinoid receptor-interacting protein 1 | Cnrip1 |
| NCALD_RAT | Neurocalcin-delta | Ncald |
| GSTM5_RAT | Glutathione S-transferase Mu 5 | Gstm5 |
| ALDOB_RAT | Fructose-bisphosphate aldolase B | Aldob |
| ANXA6_RAT | Annexin A6 | Anxa6 |
| D4A471_RAT | COX assembly mitochondrial protein | Cmc2 |
| KLH25_RAT | Kelch-like protein 25 | Klhl25 |
| DC1L2_RAT | Cytoplasmic dynein 1 light intermediate chain 2 | Dync1li2 |
| RAB6A_RAT | Ras-related protein Rab-6A | Rab6a |
| RGS18_RAT | Regulator of G-protein signaling 18 | Rgs18 |
| PP2BB_RAT | Serine/threonine-protein phosphatase 2B catalytic subunit beta isoform | Ppp3cb |
| RINI_RAT | Ribonuclease inhibitor | Rnh1 |
| IMB1_RAT | Importin subunit beta-1 | Kpnb1 |
| HPCA_RAT | Neuron-specific calcium-binding protein hippocalcin | Hpca |
| ENOB_RAT | Beta-enolase | Eno3 |
| D4A243_RAT | Enkurin domain containing 1 | Enkd1 |
| D3ZPP2_RAT | ADP-ribosylation factor like GTPase 8A | Arl8a |
| AL9A1_RAT | 4-trimethylaminobutyraldehyde dehydrogenase | Aldh9a1 |
| GSTM7_RAT | Glutathione S-transferase Mu 7 | Gstm7 |
| RAB1B_RAT | Ras-related protein Rab-1B | Rab1b |
| SCPDL_RAT | Saccharopine dehydrogenase-like oxidoreductase | Sccpdh |
| ODPB_RAT | Pyruvate dehydrogenase E1 component subunit beta, mitochondrial | Pdhb |
| CANB1_RAT | Calcineurin subunit B type 1 | Ppp3r1 |
| TBB2B_RAT | Tubulin beta-2B chain | Tubb2b |
| VAMP3_RAT | Vesicle-associated membrane protein 3 | Vamp3 |
| RMD3_RAT | Regulator of microtubule dynamics protein 3 | Rmdn3 |
| ACDSB_RAT | Short/branched chain specific acyl-CoA dehydrogenase, mitochondrial | Acadsb |
| PUR9_RAT | Bifunctional purine biosynthesis protein ATIC | Atic |
| MYPR_RAT | Myelin proteolipid protein | Plp1 |
| EF1G_RAT | Elongation factor 1-gamma | Eef1g |
| TOM70_RAT | Mitochondrial import receptor subunit TOM70 | Tomm70 |
| ARPC5_RAT | Actin-related protein 2/3 complex subunit 5 | Arpc5 |
| NAC2_RAT | Sodium/calcium exchanger 2 | Slc8a2 |
| PPM1E_RAT | Protein phosphatase 1E | Ppm1e |
| VGFR2_RAT | Vascular endothelial growth factor receptor 2 | Kdr |
| GRIA2_RAT | Glutamate receptor 2 | Gria2 |
| GSTA3_RAT | Glutathione S-transferase alpha-3 | Gsta3 |
| Q5XIV1_RAT | Phosphoglycerate kinase | Pgk2 |
| MK01_RAT | Mitogen-activated protein kinase 1 | Mapk1 |
| GBB2_RAT | Guanine nucleotide-binding protein G(I)/G(S)/G(T) subunit beta-2 | Gnb2 |
| PGFRA_RAT | Platelet-derived growth factor receptor alpha | Pdgfra |
| ODO1_RAT | 2-oxoglutarate dehydrogenase complex component E1 | Ogdh |
| PP1R7_RAT | Protein phosphatase 1 regulatory subunit 7 | Ppp1r7 |
| CALB1_RAT | Calbindin | Calb1 |
| Q8VID2_RAT | Puromycin-sensitive aminopeptidase | Npepps |
| M2OM_RAT | Mitochondrial 2-oxoglutarate/malate carrier protein | Slc25a11 |
| DHPR_RAT | Dihydropteridine reductase | Qdpr |
| PFKAL_RAT | ATP-dependent 6-phosphofructokinase, liver type | Pfkl |
| PHB2_RAT | Prohibitin-2 | Phb2 |
| NAR2B_RAT | T-cell ecto-ADP-ribosyltransferase 2 | Art2b |
| AT2A1_RAT | Sarcoplasmic/endoplasmic reticulum calcium ATPase 1 | Atp2a1 |
| SV2A_RAT | Synaptic vesicle glycoprotein 2A | Sv2a |
| CALL3_RAT | Calmodulin-like protein 3 | Calml3 |
| EAA1_RAT | Excitatory amino acid transporter 1 | Slc1a3 |
| KAD1_RAT | Adenylate kinase isoenzyme 1 | Ak1 |
| PA1B2_RAT | Platelet-activating factor acetylhydrolase IB subunit alpha2 | Pafah1b2 |
| RAB3D_RAT | GTP-binding protein Rab-3D | Rab3d |
| SAHH2_RAT | S-adenosylhomocysteine hydrolase-like protein 1 | Ahcyl1 |
| TPPP_RAT | Tubulin polymerization-promoting protein | Tppp |
| TCPA_RAT | T-complex protein 1 subunit alpha | Tcp1 |
| SIR2_RAT | NAD-dependent protein deacetylase sirtuin-2 | Sirt2 |
| VPP1_RAT | V-type proton ATPase 116 kDa subunit a 1 | Atp6v0a1 |
| TCPB_RAT | T-complex protein 1 subunit beta | Cct2 |
| HS105_RAT | Heat shock protein 105 kDa | Hsph1 |
| ADT1_RAT | ADP/ATP translocase 1 | Slc25a4 |
| CAND1_RAT | Cullin-associated NEDD8-dissociated protein 1 | Cand1 |
| VATB2_RAT | V-type proton ATPase subunit B, brain isoform | Atp6v1b2 |
| D3ZTN2_RAT | COX assembly mitochondrial protein | Cmc1 |
| D3ZCV0_RAT | Actinin alpha 2 | Actn2 |
| KCRU_RAT | Creatine kinase U-type, mitochondrial | Ckmt1 |
| DLG2_RAT | Disks large homolog 2 | Dlg2 |
| VATC1_RAT | V-type proton ATPase subunit C 1 | Atp6v1c1 |
| PFKAP_RAT | ATP-dependent 6-phosphofructokinase, platelet type | Pfkp |
| SUCA_RAT | Succinate--CoA ligase [ADP/GDP-forming] subunit alpha, mitochondrial | Suclg1 |
| AP2A2_RAT | AP-2 complex subunit alpha-2 | Ap2a2 |
| SSDH_RAT | Succinate-semialdehyde dehydrogenase, mitochondrial | Aldh5a1 |
| DPYL3_RAT | Dihydropyrimidinase-related protein 3 | Dpysl3 |
| HSP72_RAT | Heat shock-related 70 kDa protein 2 | Hspa2 |
| TERA_RAT | Transitional endoplasmic reticulum ATPase | Vcp |
| AP1B1_RAT | AP-1 complex subunit beta-1 | Ap1b1 |
| NDUS1_RAT | NADH-ubiquinone oxidoreductase 75 kDa subunit, mitochondrial | Ndufs1 |
| ODPA_RAT | Pyruvate dehydrogenase E1 component subunit alpha, somatic form, mitochondrial | Pdha1 |
| DYN1_RAT | Dynamin-1 | Dnm1 |
| KPCG_RAT | Protein kinase C gamma type | Prkcg |
| DPYL1_RAT | Dihydropyrimidinase-related protein 1 | Crmp1 |
| GSTM4_RAT | Glutathione S-transferase Mu 4 | Gstm4 |
| PP2BA_RAT | Protein phosphatase 3 catalytic subunit alpha | Ppp3ca |
| WDR1_RAT | WD repeat-containing protein 1 | Wdr1 |
| GLSK_RAT | Glutaminase kidney isoform, mitochondrial | Gls |
| NFM_RAT | Neurofilament medium polypeptide | Nefm |
| SYNJ1_RAT | Synaptojanin-1 | Synj1 |
| TBB4B_RAT | Tubulin beta-4B chain | Tubb4b |
| ACTG_RAT | Actin, cytoplasmic 2 | Actg1 |
| ATPB_RAT | ATP synthase subunit beta, mitochondrial | Atp5f1b |
| NCDN_RAT | Neurochondrin | Ncdn |
| ACTN1_RAT | Alpha-actinin-1 | Actn1 |
| AATC_RAT | Aspartate aminotransferase, cytoplasmic | Got1 |
| G3P_RAT | Glyceraldehyde-3-phosphate dehydrogenase | Gapdh |
| IDH3A_RAT | Isocitrate dehydrogenase [NAD] subunit alpha, mitochondrial | Idh3a |
| AT2B1_RAT | Plasma membrane calcium-transporting ATPase 1 | Atp2b1 |
| ACON_RAT | Aconitate hydratase, mitochondrial | Aco2 |
| ALDOA_RAT | Fructose-bisphosphate aldolase A | Aldoa |
| ADDA_RAT | Alpha-adducin | Add1 |
| MDHM_RAT | Malate dehydrogenase, mitochondrial | Mdh2 |
| LDHB_RAT | L-lactate dehydrogenase B chain | Ldhb |
| NCAM1_RAT | Neural cell adhesion molecule 1 | Ncam1 |
| HBB1_RAT | Hemoglobin subunit beta-1 | Hbb |
| CH10_RAT | 10 kDa heat shock protein, mitochondrial | Hspe1 |
| UCHL1_RAT | Ubiquitin carboxyl-terminal hydrolase isozyme L1 | Uchl1 |
| ATP5H_RAT | ATP synthase subunit d, mitochondrial | Atp5pd |
| MDHC_RAT | Malate dehydrogenase, cytoplasmic | Mdh1 |
| SYT1_RAT | Synaptotagmin-1 | Syt1 |
| THIL_RAT | Acetyl-CoA acetyltransferase, mitochondrial | Acat1 |
| S25A3_RAT | Solute carrier family 25 member 3 | Slc25a3 |
| ADT2_RAT | ADP/ATP translocase 2 | Slc25a5 |
| ATPO_RAT | ATP synthase subunit O, mitochondrial | Atp5po |
| AT1B1_RAT | Sodium/potassium-transporting ATPase subunit beta-1 | Atp1b1 |
| ATP5I_RAT | ATP synthase subunit e, mitochondrial | Atp5me |
| PRDX1_RAT | Peroxiredoxin-1 | Prdx1 |
| SEPT7_RAT | Septin-7 | Septin7 |
| SYUA_RAT | Alpha-synuclein | Snca |
| NPTN_RAT | Neuroplastin | Nptn |
| RAB3C_RAT | Ras-related protein Rab-3C | Rab3c |
| GDIR1_RAT | Rho GDP-dissociation inhibitor 1 | Arhgdia |
| MAP6_RAT | Microtubule-associated protein 6 | Map6 |
| SYGP1_RAT | Ras/Rap GTPase-activating protein SynGAP | Syngap1 |
| RAB43_RAT | Ras-related protein Rab-43 | Rab43 |
| PEA15_RAT | Astrocytic phosphoprotein PEA-15 | Pea15 |
| OXR1_RAT | Oxidation resistance protein 1 | Oxr1 |
| 1433F_RAT | 14-3-3 protein eta | Ywhah |
| D4A1W7_RAT | Nephrocystin 3 | Nphp3 |
| AT2A3_RAT | Sarcoplasmic/endoplasmic reticulum calcium ATPase 3 | Atp2a3 |
| MARCS_RAT | Myristoylated alanine-rich C-kinase substrate | Marcks |
| TCPE_RAT | T-complex protein 1 subunit epsilon | Cct5 |
| IDHP_RAT | Isocitrate dehydrogenase [NADP], mitochondrial | Idh2 |
| CLCA_RAT | Clathrin light chain A | Clta |
| H4_RAT | Histone H4 | H4c2 |
| ATPD_RAT | ATP synthase subunit delta, mitochondrial | Atp5f1d |
| SCAM5_RAT | Secretory carrier-associated membrane protein 5 | Scamp5 |
| SODC_RAT | Superoxide dismutase [Cu-Zn] | Sod1 |
| HS71A_RAT | Heat shock 70 kDa protein 1A | Hspa1a |
| B5DFG5_RAT | Septin | Septin6 |
| ARF4_RAT | ADP-ribosylation factor 4 | Arf4 |
| THIO_RAT | Thioredoxin | Txn |
| RS2_RAT | Small ribosomal subunit protein uS5 | Rps2 |
| NEC1_RAT | Neuroendocrine convertase 1 | Pcsk1 |
| D3ZKF3_RAT | MORC family CW-type zinc finger 1 | Morc1 |
| SLAP1_RAT | Src-like-adapter | Sla |
| KIF22_RAT | Kinesin-like protein KIF22 | Kif22 |
| AUHM_RAT | Methylglutaconyl-CoA hydratase, mitochondrial | Auh |
| CX7A2_RAT | Cytochrome c oxidase subunit 7A2, mitochondrial | Cox7a2 |
| MP2K2_RAT | Dual specificity mitogen-activated protein kinase kinase 2 | Map2k2 |
| GFAP_RAT | Glial fibrillary acidic protein | Gfap |
| CAH1_RAT | Carbonic anhydrase 1 | Ca1 |
| SYNPR_RAT | Synaptoporin | Synpr |
| BAIP2_RAT | Brain-specific angiogenesis inhibitor 1-associated protein 2 | Baiap2 |
| BASP1_RAT | Brain acid soluble protein 1 | Basp1 |
| WDR7_RAT | WD repeat-containing protein 7 | Wdr7 |
| MXRA8_RAT | Matrix remodeling-associated protein 8 | Mxra8 |
| SMCE1_RAT | SWI/SNF-related matrix-associated actin-dependent regulator of chromatin subfamily E member 1 | Smarce1 |
| VATL_RAT | V-type proton ATPase 16 kDa proteolipid subunit c | Atp6v0c |
| SNX20_RAT | Sorting nexin-20 | Snx20 |
| NTRI_RAT | Neurotrimin | Ntm |
| FABP7_RAT | Fatty acid-binding protein, brain | Fabp7 |
| SCAM1_RAT | Secretory carrier-associated membrane protein 1 | Scamp1 |
| KAP3_RAT | cAMP-dependent protein kinase type II-beta regulatory subunit | Prkar2b |
| RAC1_RAT | Ras-related C3 botulinum toxin substrate 1 | Rac1 |
| ANXA3_RAT | Annexin A3 | Anxa3 |
| CLD11_RAT | Claudin-11 | Cldn11 |
| EHD1_RAT | EH domain-containing protein 1 | Ehd1 |
| GMFB_RAT | Glia maturation factor beta | Gmfb |
| TXNL1_RAT | Thioredoxin-like protein 1 | Txnl1 |
| PSMD9_RAT | 26S proteasome non-ATPase regulatory subunit 9 | Psmd9 |
| CAGE1_RAT | Cancer-associated gene 1 protein homolog | Cage1 |
| MYH4_RAT | Myosin-4 | Myh4 |
| RL6_RAT | Large ribosomal subunit protein eL6 | Rpl6 |
| B1WC72_RAT | Rpgr protein | Rpgr |
| STML2_RAT | Stomatin-like protein 2, mitochondrial | Stoml2 |
| AZI2_RAT | 5-azacytidine-induced protein 2 | Azi2 |
| Q9JKY2_RAT | Zic family member 1 | Zic1 |
| B5DFM3_RAT | Ly6h protein | Ly6h |
| MYBPH_RAT | Myosin-binding protein H | Mybph |
| MTPN_RAT | Myotrophin | Mtpn |
| TBA3_RAT | Tubulin alpha-3 chain | Tuba3a |
| PCCA_RAT | Propionyl-CoA carboxylase alpha chain, mitochondrial | Pcca |
| ITIH3_RAT | Inter-alpha-trypsin inhibitor heavy chain H3 | Itih3 |
| PLD2_RAT | Phospholipase D2 | Pld2 |
| AGT2_RAT | Alanine--glyoxylate aminotransferase 2, mitochondrial | Agxt2 |
| GBRB3_RAT | Gamma-aminobutyric acid receptor subunit beta-3 | Gabrb3 |
| ACTH_RAT | Actin, gamma-enteric smooth muscle | Actg2 |
| DNJC5_RAT | DnaJ homolog subfamily C member 5 | Dnajc5 |
| B2GV82_RAT | Notchless protein homolog 1 | Nle1 |
| SNX1_RAT | Sorting nexin-1 | Snx1 |
| ZN830_RAT | Zinc finger protein 830 | Znf830 |
| Q5U1Y2_RAT | Ras-related C3 botulinum toxin substrate 2 | Rac2 |
| CRY1_RAT | Cryptochrome-1 | Cry1 |
| ARGI1_RAT | Arginase-1 | Arg1 |
| CO9_RAT | Complement component C9 | C9 |
| CA2D1_RAT | Voltage-dependent calcium channel subunit alpha-2/delta-1 | Cacna2d1 |
| DLG4_RAT | Disks large homolog 4 | Dlg4 |
| DUS1_RAT | Dual specificity protein phosphatase 1 | Dusp1 |
| F7EYR2_RAT | NOP2/Sun RNA methyltransferase family member 7 | Nsun7 |
| Q5HZY3_RAT | Ubiquitin carboxyl-terminal hydrolase | Uchl5 |
| RABE1_RAT | Rab GTPase-binding effector protein 1 | Rabep1 |
| MARK3_RAT | MAP/microtubule affinity-regulating kinase 3 | Mark3 |
| B2GUZ7_RAT | Tubulin folding cofactor C | Tbcc |
| MYH6_RAT | Myosin-6 | Myh6 |
| D3ZWC6_RAT | Syntrophin, beta 1 | Sntb1 |
| A0A0G2K9P5_RAT | Conserved oligomeric Golgi complex subunit 5 | Cog5 |
| MK08_RAT | Mitogen-activated protein kinase 8 | Mapk8 |
| NP1L4_RAT | Nucleosome assembly protein 1-like 4 | Nap1l4 |
| PAI1_RAT | Plasminogen activator inhibitor 1 | Serpine1 |
| ROA3_RAT | Heterogeneous nuclear ribonucleoprotein A3 | Hnrnpa3 |
| MPP2_RAT | MAGUK p55 subfamily member 2 | Mpp2 |
| LRC33_RAT | Transforming growth factor beta activator LRRC33 | Nrros |
| CPSF7_RAT | Cleavage and polyadenylation specificity factor subunit 7 | Cpsf7 |
| PDK1_RAT | [Pyruvate dehydrogenase | Pdk1 |
| LIPA4_RAT | Liprin-alpha-4 | Ppfia4 |
| MUG1_RAT | Murinoglobulin-1 | Mug1 |
| Q6IG11_RAT | Type II keratin Kb21 | Krt81 |
| Q6IE65_RAT | Endothelin-converting enzyme 2 | Ece2 |
| CX6C2_RAT | Cytochrome c oxidase subunit 6C-2 | Cox6c2 |
| ACTS_RAT | Actin, alpha skeletal muscle | Acta1 |
| KIF3C_RAT | Kinesin-like protein KIF3C | Kif3c |
| PUR6_RAT | Bifunctional phosphoribosylaminoimidazole carboxylase/phosphoribosylaminoimidazole succinocarboxamide synthetase | Paics |
| TXND9_RAT | Thioredoxin domain-containing protein 9 | Txndc9 |
| STX2_RAT | Syntaxin-2 | Stx2 |
| KAT8_RAT | Histone acetyltransferase KAT8 | Kat8 |
| VAT1_RAT | Synaptic vesicle membrane protein VAT-1 homolog | Vat1 |
| PGLT1_RAT | Protein O-glucosyltransferase 1 | Poglut1 |
| MYH3_RAT | Myosin-3 | Myh3 |
| RED1_RAT | Double-stranded RNA-specific editase 1 | Adarb1 |
| SYT11_RAT | Synaptotagmin-11 | Syt11 |
| PNPO_RAT | Pyridoxine-5'-phosphate oxidase | Pnpo |
| DYL2_RAT | Dynein light chain 2, cytoplasmic | Dynll2 |
| NDUS6_RAT | NADH dehydrogenase [ubiquinone] iron-sulfur protein 6, mitochondrial | Ndufs6 |
| B0K014_RAT | D-aminoacyl-tRNA deacylase | Dtd1 |
| B0K014_RAT | D-aminoacyl-tRNA deacylase | Dtd1 |
| FRMD6_RAT | FERM domain-containing protein 6 | Frmd6 |
| D4A533_RAT | Transmembrane anterior posterior transformation 1 | Tapt1 |
| SNP47_RAT | Synaptosomal-associated protein 47 | Snap47 |
| CALM1_RAT | Calmodulin-1 | Calm1 |
| KPCG_RAT | Protein kinase C gamma type | Prkcg |
| GRIA1_RAT | Glutamate receptor 1 | Gria1 |
| GRIA2_RAT | Glutamate receptor 2 | Gria2 |
| AT2B2_RAT | Plasma membrane calcium-transporting ATPase 2 | Atp2b2 |
| KCC2A_RAT | Calcium/calmodulin-dependent protein kinase type II subunit alpha | Camk2a |
| KCC2B_RAT | Calcium/calmodulin-dependent protein kinase type II subunit beta | Camk2b |
| NEUM_RAT | Neuromodulin | Gap43 |
| PARK7_RAT | Parkinson disease protein 7 homolog | Park7 |
| MAG_RAT | Myelin-associated glycoprotein | Mag |
| MBP_RAT | Myelin basic protein | Mbp |
| MOG_RAT | Myelin-oligodendrocyte glycoprotein | Mog |
| MYPR_RAT | Myelin proteolipid protein | Plp1 |
| GFAP_RAT | Glial fibrillary acidic protein | Gfap |
| DLG4_RAT | Disks large homolog 4 | Dlg4 |
| AP2B1_RAT | AP-2 complex subunit beta | Ap2b1 |
| CANB1_RAT | Calcineurin subunit B type 1 | Ppp3r1 |
| ARPC2_RAT | Actin-related protein 2/3 complex subunit 2 | Arpc2 |
| ODO1_RAT | 2-oxoglutarate dehydrogenase complex component E1 | Ogdh |
| ARC1A_RAT | Actin-related protein 2/3 complex subunit 1A | Arpc1a |
| ALDOC_RAT | Fructose-bisphosphate aldolase C | Aldoc |
| KPCD_RAT | Protein kinase C delta type | Prkcd |
| DHPR_RAT | Dihydropteridine reductase | Qdpr |
| NFM_RAT | Neurofilament medium polypeptide | Nefm |
| AATC_RAT | Aspartate aminotransferase, cytoplasmic | Got1 |
| NFL_RAT | Neurofilament light polypeptide | Nefl |
| CALB2_RAT | Calretinin | Calb2 |
| SIR2_RAT | NAD-dependent protein deacetylase sirtuin-2 | Sirt2 |
| MYO1D_RAT | Unconventional myosin-Id | Myo1d |
| AMPL_RAT | Cytosol aminopeptidase | Lap3 |
| S6A11_RAT | Sodium- and chloride-dependent GABA transporter 3 | Slc6a11 |
| UBE2N_RAT | Ubiquitin-conjugating enzyme E2 N | Ube2n |
| 1433Z_RAT | 14-3-3 protein zeta/delta | Ywhaz |
| ARF1_RAT | ADP-ribosylation factor 1 | Arf1 |
| MLP3B_RAT | Microtubule-associated proteins 1A/1B light chain 3B | Map1lc3b |
| GDIR1_RAT | Rho GDP-dissociation inhibitor 1 | Arhgdia |
| RAB18_RAT | Ras-related protein Rab-18 | Rab18 |
| B5DF65_RAT | Biliverdin reductase B | Blvrb |
| PPT1_RAT | Palmitoyl-protein thioesterase 1 | Ppt1 |
| B3VPA7_RAT | Cysteine-sulfinate decarboxylase | Csad |
| F1M1D5_RAT | Tubulin-specific chaperone D | Tbcd |
| NDKA_RAT | Nucleoside diphosphate kinase A | Nme1 |
| RAN_RAT | GTP-binding nuclear protein Ran | Ran |
| Q499Q4_RAT | Phosphoglucomutase 1 | Pgm1 |
| NCAN_RAT | Neurocan core protein | Ncan |
| ODPB_RAT | Pyruvate dehydrogenase E1 component subunit beta, mitochondrial | Pdhb |
| DHE3_RAT | Glutamate dehydrogenase 1, mitochondrial | Glud1 |
| ATPA_RAT | ATP synthase subunit alpha, mitochondrial | Atp5f1a |
| D3ZAI6_RAT | 5'-nucleotidase domain containing 3 | Nt5dc3 |
| ODP2_RAT | Dihydrolipoyllysine-residue acetyltransferase component of pyruvate dehydrogenase complex, mitochondrial | Dlat |
| F1LQ81_RAT | Vesicle-fusing ATPase | Nsf |
| VATB2_RAT | V-type proton ATPase subunit B, brain isoform | Atp6v1b2 |
| MDHM_RAT | Malate dehydrogenase, mitochondrial | Mdh2 |
| F1LN88_RAT | Acyl-CoA dehydrogenase family, member 10 | Aldh2 |
| HS90B_RAT | Heat shock protein HSP 90-beta | Hsp90ab1 |
| CX6C2_RAT | Cytochrome c oxidase subunit 6C-2 | Cox6c2 |
| HXK1_RAT | Hexokinase-1 | Hk1 |
| Q5XIH3_RAT | NADH dehydrogenase [ubiquinone] flavoprotein 1, mitochondrial | Ndufv1 |
| A0A097BVJ5_RAT | 2',3'-cyclic-nucleotide 3'-phosphodiesterase | Cnp |
| F7FKI5_RAT | Pyruvate dehydrogenase E1 component subunit alpha | Pdha1 |
| GNAO_RAT | Guanine nucleotide-binding protein G(o) subunit alpha | Gnao1 |
| AT1A2_RAT | Sodium/potassium-transporting ATPase subunit alpha-2 | Atp1a2 |
| G3V6P2_RAT | Dihydrolipoyllysine-residue succinyltransferase component of 2-oxoglutarate dehydrogenase complex, mitochondrial | Dlst |
| F1M953_RAT | Stress-70 protein, mitochondrial | Hspa9 |
| B2GV33_RAT | Amine oxidase | Maoa |
| B2RYT5_RAT | Cox7a2l protein | Cox7a2l |
| NDUS1_RAT | NADH-ubiquinone oxidoreductase 75 kDa subunit, mitochondrial | Ndufs1 |
| VDAC2_RAT | Voltage-dependent anion-selective channel protein 2 | Vdac2 |
| VDAC1_RAT | Voltage-dependent anion-selective channel protein 1 | Vdac1 |
| F1LQJ7_RAT | phosphoenolpyruvate carboxykinase | Pck2 |
| A0A0G2JTL5_RAT | pyruvate carboxylase | Pc |
| ADT2_RAT | ADP/ATP translocase 2 | Slc25a5 |
| I1T7F1_RAT | Amino acid transporter | N/A |
| F1LPG5_RAT | NADH dehydrogenase [ubiquinone] 1 beta subcomplex subunit 4 | Ndufb4 |
| AOFB_RAT | Amine oxidase [flavin-containing] B | Maob |
| KCC2A_RAT | Calcium/calmodulin-dependent protein kinase type II subunit alpha | Camk2a |
| SFXN5_RAT | Sideroflexin-5 | Sfxn5 |
| COX41_RAT | Cytochrome c oxidase subunit 4 isoform 1, mitochondrial | Cox4i1 |
| G3V945_RAT | Succinate-semialdehyde dehydrogenase | Aldh5a1 |
| ATPG_RAT | ATP synthase subunit gamma, mitochondrial | Atp5f1c |
| Q6P9Y4_RAT | ADP/ATP translocase | Slc25a4 |
| S2512_RAT | Electrogenic aspartate/glutamate antiporter SLC25A12, mitochondrial | Slc25a12 |
| Q2I6B2_RAT | V-type proton ATPase subunit a | Atp6v0a1 |
| ACON_RAT | Aconitate hydratase, mitochondrial | Aco2 |
| G3V741_RAT | Phosphate carrier protein, mitochondrial | Slc25a3 |
| A0A0G2JVH4_RAT | MICOS complex subunit MIC60 | Immt |
| ATPD_RAT | ATP synthase subunit delta, mitochondrial | Atp5f1d |
| COX5A_RAT | Cytochrome c oxidase subunit 5A, mitochondrial | Cox5a |
| DLDH_RAT | Dihydrolipoyl dehydrogenase, mitochondrial | Dld |
| B2GUZ3_RAT | formate--tetrahydrofolate ligase | Mthfd1l |
| Q5RK08_RAT | Glioblastoma amplified sequence | Nipsnap2 |
| D3ZE15_RAT | NADH dehydrogenase [ubiquinone] 1 alpha subcomplex subunit 13 | Ndufa13 |
| AT5F1_RAT | ATP synthase F(0) complex subunit B1, mitochondrial | Atp5pb |
| A0A0G2KB63_RAT | Prohibitin | Phb2 |
| PHB1_RAT | Prohibitin 1 | Phb1 |
| B2RZ24_RAT | Succinate-CoA ligase subunit beta | Sucla2 |
| G3V936_RAT | Citrate synthase | Cs |
| ODO1_RAT | 2-oxoglutarate dehydrogenase complex component E1 | Ogdh |
| CYC_RAT | Cytochrome c, somatic | Cycs |
| AATM_RAT | Aspartate aminotransferase, mitochondrial | Got2 |
| QCR2_RAT | Cytochrome b-c1 complex subunit 2, mitochondrial | Uqcrc2 |
| G3V6D3_RAT | ATP synthase subunit beta | Atp5f1b |
| GHC1_RAT | Mitochondrial glutamate carrier 1 | Slc25a22 |
| ATPO_RAT | ATP synthase subunit O, mitochondrial | Atp5po |
| SUCA_RAT | Succinate--CoA ligase [ADP/GDP-forming] subunit alpha, mitochondrial | Suclg1 |
| G3V6H5_RAT | Solute carrier family 25 member 11 | Slc25a11 |
| D3ZG43_RAT | NADH dehydrogenase [ubiquinone] iron-sulfur protein 3, mitochondrial | Ndufs3 |
| GLNA_RAT | Glutamine synthetase | Glul |
| CH60_RAT | 60 kDa heat shock protein, mitochondrial | Hspd1 |
| CPLX2_RAT | Complexin-2 | Cplx2 |
| ARL1_RAT | ADP-ribosylation factor-like protein 1 | Arl1 |
| MIF_RAT | Macrophage migration inhibitory factor | Mif |
| Q6AYD5_RAT | G1 to S phase transition 1 | Gspt1 |
| Q6IE67_RAT | Proteasome subunit alpha type | LOC100361067 |
| A0A0G2KAT4_RAT | DExD-box helicase 39B | Ddx39b |
| Q5U2S7_RAT | Proteasome 26S subunit, non-ATPase 3 | Psmd3 |
| D3ZHB7_RAT | HECT-type E3 ubiquitin transferase | Ube3c |
| SODC_RAT | Superoxide dismutase [Cu-Zn] | Sod1 |
| KPYM_RAT | Pyruvate kinase PKM | Pkm |
| Q3MHS9_RAT | Chaperonin containing Tcp1, subunit 6A | Cct6a |
| A0A0G2JSQ1_RAT | Beta-synuclein | Sncb |
| G3V7G9_RAT | Eukaryotic translation initiation factor 3 subunit L | Eif3l |
| A0A0G2K0F3_RAT | Erythrocyte membrane protein band 4.1-like 1 | Epb41l1 |
| S100B_RAT | Protein S100-B | S100b |
| LYPA1_RAT | Acyl-protein thioesterase 1 | Lypla1 |
| WDR1_RAT | WD repeat-containing protein 1 | Wdr1 |
| G3V7C6_RAT | Tubulin beta chain | Tubb4b |
| KPYM_RAT | Pyruvate kinase PKM | Pkm |
| DCE2_RAT | Glutamate decarboxylase 2 | Gad2 |
| TCPD_RAT | T-complex protein 1 subunit delta | Cct4 |
| A0A0G2JT00_RAT | CutA divalent cation tolerance homolog | Cuta |
| D3ZDT1_RAT | Erythrocyte membrane protein band 4.1-like 2 | Epb41l2 |
| RIPR1_RAT | Rho family-interacting cell polarization regulator 1 | Ripor1 |
| TBB5_RAT | Tubulin beta-5 chain | Tubb5 |
| UBA1_RAT | Ubiquitin-like modifier-activating enzyme 1 | Uba1 |
| B4F7C2_RAT | Tubulin beta chain | Tubb4a |
| SAHH2_RAT | S-adenosylhomocysteine hydrolase-like protein 1 | Ahcyl1 |
| PARK7_RAT | Parkinson disease protein 7 homolog | Park7 |
| D3Z955_RAT | Phosphoglucomutase 2-like 1 | Pgm2l1 |
| HINT1_RAT | Adenosine 5'-monophosphoramidase HINT1 | Hint1 |
| UCHL1_RAT | Ubiquitin carboxyl-terminal hydrolase isozyme L1 | Uchl1 |
| D4A4U3_RAT | Magnesium-dependent phosphatase 1 | Mdp1 |
| DDAH1_RAT | N(G),N(G)-dimethylarginine dimethylaminohydrolase 1 | Ddah1 |
| PIMT_RAT | Protein-L-isoaspartate(D-aspartate) O-methyltransferase | Pcmt1 |
| IDHC_RAT | Isocitrate dehydrogenase [NADP] cytoplasmic | Idh1 |
| D4AC23_RAT | T-complex protein 1 subunit eta | Cct7 |
| D4AEH3_RAT | Proteasome 26S subunit, non-ATPase 7 | Psmd7 |
| GFAP_RAT | Glial fibrillary acidic protein | Gfap |
| TBB2A_RAT | Tubulin beta-2A chain | Tubb2a |
| SYSC_RAT | Serine--tRNA ligase, cytoplasmic | Sars1 |
| D3ZXS8_RAT | Ubiquitin-conjugating enzyme E2K | Ube2k |
| GSH1_RAT | Glutamate--cysteine ligase catalytic subunit | Gclc |
| CSPG2_RAT | Versican core protein | Vcan |
| A0A0G2JW38_RAT | Ribulose-phosphate 3-epimerase | Rpe |
| A0A0G2K1Q9_RAT | Erythrocyte membrane protein band 4.1-like 3 | Epb41l3 |
| PEA15_RAT | Astrocytic phosphoprotein PEA-15 | Pea15 |
| PPM1E_RAT | Protein phosphatase 1E | Ppm1e |
| A0A0G2K9L2_RAT | Target of myb1 like 2 membrane trafficking protein | Tom1l2 |
| G3V8B6_RAT | 26S proteasome non-ATPase regulatory subunit 1 | Psmd1 |
| I7FKL4_RAT | Myelin basic protein | Mbp |
| SYUA_RAT | Alpha-synuclein | Snca |
| GDIA_RAT | Rab GDP dissociation inhibitor alpha | Gdi1 |
| PGTA_RAT | Geranylgeranyl transferase type-2 subunit alpha | Rabggta |
| LIPA3_RAT | Liprin-alpha-3 | Ppfia3 |
| B4F772_RAT | Heat shock 70kDa protein 4-like | Hspa4l |
| D4AA63_RAT | Ubiquilin 2 | Ubqln2 |
| PSD11_RAT | 26S proteasome non-ATPase regulatory subunit 11 | Psmd11 |
| KIF5C_RAT | Kinesin heavy chain isoform 5C | Kif5c |
| PSA1_RAT | Proteasome subunit alpha type-1 | Psma1 |
| COMT_RAT | Catechol O-methyltransferase | Comt |
| KAD1_RAT | Adenylate kinase isoenzyme 1 | Ak1 |
| NISCH_RAT | Nischarin | Nisch |
| STIP1_RAT | Stress-induced-phosphoprotein 1 | Stip1 |
| NCDN_RAT | Neurochondrin | Ncdn |
| CSN8_RAT | COP9 signalosome complex subunit 8 | Cops8 |
| OTUB1_RAT | Ubiquitin thioesterase OTUB1 | Otub1 |
| PDXK_RAT | Pyridoxal kinase | Pdxk |
| HSP7C_RAT | Heat shock cognate 71 kDa protein | Hspa8 |
| Q3ZAU6_RAT | RBR-type E3 ubiquitin transferase | Rnf14 |
| D4A6C5_RAT | Rho GTPase activating protein 1 | Arhgap1 |
| F1LPP0_RAT | Amphiphysin-like | Amph |
| F1M951_RAT | Protein tyrosine phosphatase, non-receptor type 23 | Ptpn23 |
| Q3MID5_RAT | Phosphatidylinositol-4-phosphate 5-kinase, type I, gamma | Pip5k1c |
| Q52KS1_RAT | ATP-dependent 6-phosphofructokinase | Pfkm |
| VCIP1_RAT | Deubiquitinating protein VCPIP1 | Vcpip1 |
| SH3G3_RAT | Endophilin-A3 | Sh3gl3 |
| SCG2_RAT | Secretogranin-2 | Scg2 |
| PPT1_RAT | Palmitoyl-protein thioesterase 1 | Ppt1 |
| TMOD2_RAT | Tropomodulin-2 | Tmod2 |
| EIF3J_RAT | Eukaryotic translation initiation factor 3 subunit J | Eif3j |
| CADM1_RAT | Cell adhesion molecule 1 | Cadm1 |
| SCG1_RAT | Secretogranin-1 | Chgb |
| CPLX1_RAT | Complexin-1 | Cplx1 |
| CPLX2_RAT | Complexin-2 | Cplx2 |
| MTAP2_RAT | Microtubule-associated protein 2 | Map2 |
| NUCL_RAT | Nucleolin | Ncl |
| SRSF2_RAT | Serine/arginine-rich splicing factor 2 | Srsf2 |
| VCIP1_RAT | Deubiquitinating protein VCPIP1 | Vcpip1 |
| EXOC8_RAT | Exocyst complex component 8 | Exoc8 |
| CSDC2_RAT | Cold shock domain-containing protein C2 | Csdc2 |
| NECA1_RAT | N-terminal EF-hand calcium-binding protein 1 | Necab1 |
| CD47_RAT | Leukocyte surface antigen CD47 | Cd47 |
| MYPR_RAT | Myelin proteolipid protein | Plp1 |
| AT1A1_RAT | Sodium/potassium-transporting ATPase subunit alpha-1 | Atp1a1 |
| AT2B4_RAT | Plasma membrane calcium-transporting ATPase 4 | Atp2b4 |
| AT1B1_RAT | Sodium/potassium-transporting ATPase subunit beta-1 | Atp1b1 |
| AT1A3_RAT | Sodium/potassium-transporting ATPase subunit alpha-3 | Atp1a3 |
| THY1_RAT | Thy-1 membrane glycoprotein | Thy1 |
| THIKA_RAT | 3-ketoacyl-CoA thiolase A, peroxisomal | Acaa1a |
| NPTN_RAT | Neuroplastin | Nptn |
| GPM6A_RAT | Neuronal membrane glycoprotein M6-a | Gpm6a |
| SHPS1_RAT | Tyrosine-protein phosphatase non-receptor type substrate 1 | Sirpa |
| RAP1B_RAT | Ras-related protein Rap-1b | Rap1b |
| AT2B2_RAT | Plasma membrane calcium-transporting ATPase 2 | Atp2b2 |
| KBP_RAT | KIF-binding protein | Kifbp |
| OPCM_RAT | Opioid-binding protein/cell adhesion molecule | Opcml |
| CA2D1_RAT | Voltage-dependent calcium channel subunit alpha-2/delta-1 | Cacna2d1 |
| NTRI_RAT | Neurotrimin | Ntm |
| ORN_RAT | Oligoribonuclease, mitochondrial | Rexo2 |
| IGG2A_RAT | Ig gamma-2A chain C region | Igg-2a |
| NCAM1_RAT | Neural cell adhesion molecule 1 | Ncam1 |
| MPPA_RAT | Mitochondrial-processing peptidase subunit alpha | Pmpca |
| NRX3A_RAT | Neurexin-3 | Nrxn3 |
| HMCS1_RAT | Hydroxymethylglutaryl-CoA synthase, cytoplasmic | Hmgcs1 |
| RAB5A_RAT | Ras-related protein Rab-5A | Rab5a |
| MDHM_RAT | Malate dehydrogenase, mitochondrial | Mdh2 |
| PLD3_RAT | 5'-3' exonuclease PLD3 | Pld3 |
| SODM_RAT | Superoxide dismutase [Mn], mitochondrial | Sod2 |
| MVD1_RAT | Diphosphomevalonate decarboxylase | Mvd |
| GNAI1_RAT | Guanine nucleotide-binding protein G(i) subunit alpha-1 | Gnai1 |
| HYOU1_RAT | Hypoxia up-regulated protein 1 | Hyou1 |
| GNAO_RAT | Guanine nucleotide-binding protein G(o) subunit alpha | Gnao1 |
| ENPL_RAT | Endoplasmin | Hsp90b1 |
| BIP_RAT | Endoplasmic reticulum chaperone BiP | Hspa5 |
| RASH_RAT | GTPase HRas | Hras |
| UCRI_RAT | Cytochrome b-c1 complex subunit Rieske, mitochondrial | Uqcrfs1 |
| NAC2_RAT | Sodium/calcium exchanger 2 | Slc8a2 |
| RAP2B_RAT | Ras-related protein Rap-2b | Rap2b |
| THIM_RAT | 3-ketoacyl-CoA thiolase, mitochondrial | Acaa2 |
| TOM34_RAT | Mitochondrial import receptor subunit TOM34 | Tomm34 |
| GNAO_RAT | Guanine nucleotide-binding protein G(o) subunit alpha | Gnao1 |
| GNAS2_RAT | Guanine nucleotide-binding protein G(s) subunit alpha isoforms short | Gnas |
| GNAI2_RAT | Guanine nucleotide-binding protein G(i) subunit alpha-2 | Gnai2 |
| A0A0G2JTA1_RAT | Serine/threonine-protein phosphatase 2A 56 kDa regulatory subunit | Ppp2r5e |
| TBB2A_RAT | Tubulin beta-2A chain | Tubb2a |
| G3V6D3_RAT | ATP synthase subunit beta | Atp5f1b |
| A0A0G2K9J2_RAT | V-type proton ATPase subunit H | Atp6v1h |
| AP2B1_RAT | AP-2 complex subunit beta | Ap2b1 |
| TCPB_RAT | T-complex protein 1 subunit beta | Cct2 |
| PP2BA_RAT | Protein phosphatase 3 catalytic subunit alpha | Ppp3ca |
| ACTN1_RAT | Alpha-actinin-1 | Actn1 |
| ATPA_RAT | ATP synthase subunit alpha, mitochondrial | Atp5f1a |
| PRS7_RAT | 26S proteasome regulatory subunit 7 | Psmc2 |
| F8WFM2_RAT | NSF attachment protein beta | Napb |
| NCDN_RAT | Neurochondrin | Ncdn |
| ACBG1_RAT | Long-chain-fatty-acid--CoA ligase ACSBG1 | Acsbg1 |
| DYN1_RAT | Dynamin-1 | Dnm1 |
| CLH1_RAT | Clathrin heavy chain 1 | Cltc |
| F1M779_RAT | Clathrin heavy chain | Cltc |
| Q499Q4_RAT | Phosphoglucomutase 1 | Pgm1 |
| GABT_RAT | 4-aminobutyrate aminotransferase, mitochondrial | Abat |
| D3ZC55_RAT | Heat shock protein family A | Hspa12a |
| KAT3_RAT | Kynurenine--oxoglutarate transaminase 3 | Kyat3 |
| A0A0G2JSM7_RAT | Adducin 1 | Add1 |
| SYT1_RAT | Synaptotagmin-1 | Syt1 |
| Q9JMG8_RAT | Dihydropyrimidinase-related protein | N/A |
| GLSK_RAT | Glutaminase kidney isoform, mitochondrial | Gls |
| F1M3W5_RAT | Dmx-like 2 | Dmxl2 |
| F8WFW5_RAT | Phosphodiesterase | Pde2a |
| FAHD2_RAT | Fumarylacetoacetate hydrolase domain-containing protein 2 | Fahd2 |
| F1LML2_RAT | Ubiquitin C | Ubc |
| Q9JKB7_RAT | Guanine deaminase | Gda |
| H1UBM5_RAT | Copine 6 protein | Cpne6 |
| HS105_RAT | Heat shock protein 105 kDa | Hsph1 |
| AATM_RAT | Aspartate aminotransferase, mitochondrial | Got2 |
| D3ZUY8_RAT | AP-2 complex subunit alpha | Ap2a1 |
| SHLB2_RAT | Endophilin-B2 | Sh3glb2 |
| IF4A2_RAT | Eukaryotic initiation factor 4A-II | Eif4a2 |
| Q3MHS9_RAT | Chaperonin containing Tcp1, subunit 6A | Cct6a |
| ENOG_RAT | Gamma-enolase | Eno2 |
| ODO1_RAT | 2-oxoglutarate dehydrogenase complex component E1 | Ogdh |
| SH3G2_RAT | Endophilin-A1 | Sh3gl2 |
| GDIA_RAT | Rab GDP dissociation inhibitor alpha | Gdi1 |
| CAP2_RAT | Adenylyl cyclase-associated protein 2 | Cap2 |
| G3V7I5_RAT | Aldehyde dehydrogenase 1 family, member B1 | Aldh1b1 |
| A0A0G2K7Y2_RAT | Oxidation resistance 1 | Oxr1 |
| NDRG2_RAT | Protein NDRG2 | Ndrg2 |
| B2GV33_RAT | Amine oxidase | Maoa |
| PLCB1_RAT | 1-phosphatidylinositol 4,5-bisphosphate phosphodiesterase beta-1 | Plcb1 |
| G3V7L8_RAT | ATPase H+ transporting V1 subunit E1 | Atp6v1e1 |
| BIP_RAT | Endoplasmic reticulum chaperone BiP | Hspa5 |
| KPYM_RAT | Pyruvate kinase PKM | Pkm |
| Q5XIH3_RAT | NADH dehydrogenase [ubiquinone] flavoprotein 1, mitochondrial | Ndufv1 |
| B2RYG2_RAT | phosphoenolpyruvate carboxykinase | Pck2 |
| UBA1_RAT | Ubiquitin-like modifier-activating enzyme 1 | Uba1 |
| 1433E_RAT | 14-3-3 protein epsilon | Ywhae |
| B4F772_RAT | Heat shock 70kDa protein 4-like | Hspa4l |
| DPYL3_RAT | Dihydropyrimidinase-related protein 3 | Dpysl3 |
| A0A0G2JZH8_RAT | Dihydrolipoamide acetyltransferase component of pyruvate dehydrogenase complex | Pdhx |
| G3V6Y6_RAT | Alpha-1,4 glucan phosphorylase | Pygb |
| Q5XI34_RAT | Protein phosphatase 2 scaffold subunit A alpha | Ppp2r1a |
| SERA_RAT | D-3-phosphoglycerate dehydrogenase | Phgdh |
| F1LQ81_RAT | Vesicle-fusing ATPase | Nsf |
| HSP7C_RAT | Heat shock cognate 71 kDa protein | Hspa8 |
| F1LRV4_RAT | Heat shock 70 kDa protein 4 | Hspa4 |
| HS90A_RAT | Heat shock protein HSP 90-alpha | Hsp90aa1 |
| DYL1_RAT | Dynein light chain 1, cytoplasmic | Dynll1 |
| EF1G_RAT | Elongation factor 1-gamma | Eef1g |
| MMSA_RAT | Methylmalonate-semialdehyde dehydrogenase [acylating], mitochondrial | Aldh6a1 |
| F1LQ63_RAT | Tenascin R | Tnr |
| D4A133_RAT | H(+)-transporting two-sector ATPase | Atp6v1a |
| D3ZAI6_RAT | 5'-nucleotidase domain containing 3 | Nt5dc3 |
| F1M9V7_RAT | Aminopeptidase | Npepps |
| LDHB_RAT | L-lactate dehydrogenase B chain | Ldhb |
| CH60_RAT | 60 kDa heat shock protein, mitochondrial | Hspd1 |
| PEBP1_RAT | Phosphatidylethanolamine-binding protein 1 | Pebp1 |
| MDHC_RAT | Malate dehydrogenase, cytoplasmic | Mdh1 |
| D4A435_RAT | Intercellular adhesion molecule 5 | Icam5 |
| ALDOA_RAT | Fructose-bisphosphate aldolase A | Aldoa |
| RAB3A_RAT | Ras-related protein Rab-3A | Rab3a |
| A0A0G2JZ69_RAT | Spectrin, alpha, non-erythrocytic 1 | Sptan1 |
| F1LMY3_RAT | protein-tyrosine-phosphatase | Ptprz1 |
| OPCM_RAT | Opioid-binding protein/cell adhesion molecule | Opcml |
| SCOT1_RAT | Succinyl-CoA:3-ketoacid coenzyme A transferase 1, mitochondrial | Oxct1 |
| Q5EB49_RAT | phosphopyruvate hydratase | Eno1 |
| DPYL2_RAT | Dihydropyrimidinase-related protein 2 | Dpysl2 |
| PROF2_RAT | Profilin-2 | Pfn2 |
| MAP6_RAT | Microtubule-associated protein 6 | Map6 |
| CH10_RAT | 10 kDa heat shock protein, mitochondrial | Hspe1 |
| D3ZS58_RAT | NADH dehydrogenase [ubiquinone] 1 alpha subcomplex subunit 2 | Ndufa2 |
| EAA2_RAT | Excitatory amino acid transporter 2 | Slc1a2 |
| MYPR_RAT | Myelin proteolipid protein | Plp1 |
| ATP5I_RAT | ATP synthase subunit e, mitochondrial | Atp5me |
| AKAP5_RAT | A-kinase anchor protein 5 | Akap5 |
| TCPD_RAT | T-complex protein 1 subunit delta | Cct4 |
| EFTU_RAT | Elongation factor Tu, mitochondrial | Tufm |
| TPPP_RAT | Tubulin polymerization-promoting protein | Tppp |
| DCTN2_RAT | Dynactin subunit 2 | Dctn2 |
| MAP1B_RAT | Microtubule-associated protein 1B | Map1b |
| GBB2_RAT | Guanine nucleotide-binding protein G(I)/G(S)/G(T) subunit beta-2 | Gnb2 |
| RAP1A_RAT | Ras-related protein Rap-1A | Rap1a |
| RASK_RAT | GTPase KRas | Kras |
| BAIP2_RAT | Brain-specific angiogenesis inhibitor 1-associated protein 2 | Baiap2 |
| SEPT9_RAT | Septin-9 | Septin9 |
| MAP1A_RAT | Microtubule-associated protein 1A | Map1a |
| CLH1_RAT | Clathrin heavy chain 1 | Cltc |
| LRP1_RAT | Prolow-density lipoprotein receptor-related protein 1 | Lrp1 |
| CATA_RAT | Catalase | Cat |
| KAPCB_RAT | cAMP-dependent protein kinase catalytic subunit beta | Prkacb |
| TBA4A_RAT | Tubulin alpha-4A chain | Tuba4a |
| LRC57_RAT | Leucine-rich repeat-containing protein 57 | Lrrc57 |
| SNAA_RAT | Alpha-soluble NSF attachment protein | Napa |
| TBB5_RAT | Tubulin beta-5 chain | Tubb5 |
| SYPH_RAT | Synaptophysin | Syp |
| MYH10_RAT | Myosin-10 | Myh10 |
| NAC2_RAT | Sodium/calcium exchanger 2 | Slc8a2 |
| AT1B2_RAT | Sodium/potassium-transporting ATPase subunit beta-2 | Atp1b2 |
| ACY2_RAT | Aspartoacylase | Aspa |
| CALX_RAT | Calnexin | Canx |
| COX5B_RAT | Cytochrome c oxidase subunit 5B, mitochondrial | Cox5b |
| HOT_RAT | Hydroxyacid-oxoacid transhydrogenase, mitochondrial | Adhfe1 |
| IGG2A_RAT | Ig gamma-2A chain C region | Igg-2a |
| HBA_RAT | Hemoglobin subunit alpha-1/2 | Hba1 |
| 6PGD_RAT | 6-phosphogluconate dehydrogenase, decarboxylating | Pgd |
| MDHM_RAT | Malate dehydrogenase, mitochondrial | Mdh2 |
| SODC_RAT | Superoxide dismutase [Cu-Zn] | Sod1 |

| **Supplemental Table 4.** The results of drug-associated metabolite set enrichment analysis. | | | | |
| --- | --- | --- | --- | --- |
| **Drug-associated metabolite sets** | **Hits** | **Total** | ***P* value** | **FDR** |
| Disulfiram action pathway | 14 | 76 | < 0.001 | < 0.001 |
| Mercaptopurine action pathway | 13 | 86 | < 0.001 | 0.001 |
| Thioguanine action pathway | 13 | 87 | < 0.001 | 0.001 |
| Azathioprine action pathway | 13 | 88 | < 0.001 | 0.001 |
| Carfentanil action pathway | 3 | 10 | 0.005 | 0.042 |
| Amikacin action pathway | 4 | 20 | 0.006 | 0.042 |
| Arbekacin action pathway | 4 | 20 | 0.006 | 0.042 |
| Azithromycin action pathway | 4 | 20 | 0.006 | 0.042 |
| Chloramphenicol action pathway | 4 | 20 | 0.006 | 0.042 |
| Clarithromycin action pathway | 4 | 20 | 0.006 | 0.042 |
| Clindamycin action pathway | 4 | 20 | 0.006 | 0.042 |
| Clomocycline action pathway | 4 | 20 | 0.006 | 0.042 |
| Demeclocycline action pathway | 4 | 20 | 0.006 | 0.042 |
| Doxycycline action pathway | 4 | 20 | 0.006 | 0.042 |
| Erythromycin action pathway | 4 | 20 | 0.006 | 0.042 |
| Gentamicin action pathway | 4 | 20 | 0.006 | 0.042 |
| Josamycin action pathway | 4 | 20 | 0.006 | 0.042 |
| Kanamycin action pathway | 4 | 20 | 0.006 | 0.042 |
| Lincomycin action pathway | 4 | 20 | 0.006 | 0.042 |
| Lymecycline action pathway | 4 | 20 | 0.006 | 0.042 |
| Methacycline action pathway | 4 | 20 | 0.006 | 0.042 |
| Minocycline action pathway | 4 | 20 | 0.006 | 0.042 |
| Neomycin action pathway | 4 | 20 | 0.006 | 0.042 |
| Netilmicin action pathway | 4 | 20 | 0.006 | 0.042 |
| Oxytetracycline action pathway | 4 | 20 | 0.006 | 0.042 |
| Paromomycin action pathway | 4 | 20 | 0.006 | 0.042 |
| Rolitetracycline action pathway | 4 | 20 | 0.006 | 0.042 |
| Roxithromycin action pathway | 4 | 20 | 0.006 | 0.042 |
| Spectinomycin action pathway | 4 | 20 | 0.006 | 0.042 |
| Streptomycin action pathway | 4 | 20 | 0.006 | 0.042 |
| Telithromycin action pathway | 4 | 20 | 0.006 | 0.042 |
| Tetracycline action pathway | 4 | 20 | 0.006 | 0.042 |
| Tigecycline action pathway | 4 | 20 | 0.006 | 0.042 |
| Tobramycin action pathway | 4 | 20 | 0.006 | 0.042 |
| Troleandomycin action pathway | 4 | 20 | 0.006 | 0.042 |
| 3-methylthiofentanyl action pathway | 3 | 11 | 0.007 | 0.042 |
| Alfentanil action pathway | 3 | 11 | 0.007 | 0.042 |
| Alvimopan action pathway | 3 | 11 | 0.007 | 0.042 |
| Anileridine action pathway | 3 | 11 | 0.007 | 0.042 |
| Benzocaine action pathway | 3 | 11 | 0.007 | 0.042 |
| Bupivacaine action pathway | 3 | 11 | 0.007 | 0.042 |
| Buprenorphine action pathway | 3 | 11 | 0.007 | 0.042 |
| Chloroprocaine action pathway | 3 | 11 | 0.007 | 0.042 |
| Cocaine action pathway | 3 | 11 | 0.007 | 0.042 |
| Dezocine action pathway | 3 | 11 | 0.007 | 0.042 |
| Dibucaine action pathway | 3 | 11 | 0.007 | 0.042 |
| Dihydromorphine action pathway | 3 | 11 | 0.007 | 0.042 |
| Dimethylthiambutene action pathway | 3 | 11 | 0.007 | 0.042 |
| Diphenoxylate action pathway | 3 | 11 | 0.007 | 0.042 |
| Escitalopram action pathway | 3 | 11 | 0.007 | 0.042 |
| Ethylmorphine action pathway | 3 | 11 | 0.007 | 0.042 |
| Fentanyl action pathway | 3 | 11 | 0.007 | 0.042 |
| Hydrocodone action pathway | 3 | 11 | 0.007 | 0.042 |
| Hydromorphone action pathway | 3 | 11 | 0.007 | 0.042 |
| Ketobemidone action pathway | 3 | 11 | 0.007 | 0.042 |
| Levallorphan action pathway | 3 | 11 | 0.007 | 0.042 |
| Levobupivacaine action pathway | 3 | 11 | 0.007 | 0.042 |
| Levomethadyl acetate action action pathway | 3 | 11 | 0.007 | 0.042 |
| Levorphanol action pathway | 3 | 11 | 0.007 | 0.042 |
| Mepivacaine action pathway | 3 | 11 | 0.007 | 0.042 |
| Methadyl acetate action pathway | 3 | 11 | 0.007 | 0.042 |
| Nalbuphine action pathway | 3 | 11 | 0.007 | 0.042 |
| Naloxone action pathway | 3 | 11 | 0.007 | 0.042 |
| Naltrexone action pathway | 3 | 11 | 0.007 | 0.042 |
| Oxybuprocaine action pathway | 3 | 11 | 0.007 | 0.042 |
| Oxycodone action pathway | 3 | 11 | 0.007 | 0.042 |
| Oxymorphone action pathway | 3 | 11 | 0.007 | 0.042 |
| Pentazocine action pathway | 3 | 11 | 0.007 | 0.042 |
| Prilocaine action pathway | 3 | 11 | 0.007 | 0.042 |
| Procaine action pathway | 3 | 11 | 0.007 | 0.042 |
| Proparacaine action pathway | 3 | 11 | 0.007 | 0.042 |
| Propoxyphene action pathway | 3 | 11 | 0.007 | 0.042 |
| Remifentanil action pathway | 3 | 11 | 0.007 | 0.042 |
| Ropivacaine action pathway | 3 | 11 | 0.007 | 0.042 |
| Sufentanil action pathway | 3 | 11 | 0.007 | 0.042 |
| Tramadol action action pathway | 3 | 11 | 0.007 | 0.042 |
| Heroin action pathway | 3 | 13 | 0.012 | 0.068 |
| Morphine action pathway | 3 | 16 | 0.021 | 0.121 |
| Desipramine action pathway | 3 | 18 | 0.029 | 0.163 |
| Methadone action pathway | 3 | 18 | 0.029 | 0.163 |
| Fluoxetine action pathway | 3 | 19 | 0.034 | 0.186 |
| Codeine action pathway | 3 | 21 | 0.044 | 0.240 |
| Imipramine action pathway | 3 | 22 | 0.050 | 0.267 |
| Note: Hits, the number of genes that are successfully mapped to a specific pathway; *P* values were calculated from hypergeometric tests, and false discovery rates (FDRs) were then calculated; Total, the total number of all genes contained in the pathway. | | | | |
